# Functional Characterization of Deubiquitinase UBP Family and Proteomic Analysis of Aaubp14-Mediated Pathogenicity Mechanism in *Alternaria alternata*

**DOI:** 10.3390/jof11070495

**Published:** 2025-06-29

**Authors:** Jiejing Tang, Hang Zhou, Chen Jiao, Hongye Li

**Affiliations:** 1 The Key Laboratory of Biology of Crop Pathogens and Insects of Zhejiang Province, The Key Laboratory of Molecular Biology of Crop Pathogens and Insects of Ministry of Agriculture, Institute of Biotechnology, Zhejiang University, Hangzhou 310058, China; tangjiejing@zju.edu.cn (J.T.); biochenjiao@zju.edu.cn (C.J.); 2 The Key Laboratory of Biology of Crop Pathogens and Insects of Zhejiang Province, Institute of Insect Science, Zhejiang University, Hangzhou 310058, China; zhouhang716@zju.edu.cn

**Keywords:** *Alternaria alternata* tangerine pathotype, ubiquitin-specific protease, ACT biosynthesis, pathogenicity, ubiquitome

## Abstract

The *Alternaria alternata* tangerine pathotype causes Alternaria brown spot, a devastating disease of susceptible tangerine varieties and their hybrids. *Alternaria citri* toxin (ACT) is the primary virulence factor, but the regulatory mechanisms governing ACT synthesis remain unclear. Deubiquitinating enzymes maintain ubiquitination homeostasis and regulate fungal pathogenicity, yet their role in *A. alternata* remains unexplored. We characterized 13 ubiquitin-specific protease (UBP) family members in *A. alternata* tangerine pathotype. Six UBP genes (*Aaubp2*, *Aaubp3*, *Aaubp4*, *Aaubp6*, *Aaubp14*, and *Aaubp15*) regulated mycelial growth. *Aaubp14* deletion abolished sporulation, while mutations of *Aaubp3*, *Aaubp4*, *Aaubp6*, *Aaubp8*, and *Aaubp15* altered conidial morphology. qRT-PCR demonstrated distinct host-induced expression patterns among *Aaubp* genes. Pathogenicity tests showed that Δ*Aaubp6*, Δ*Aaubp14*, and Δ*Aaubp15* mutants failed to produce lesions on *Citrus reticulata* cv. Hongjv leaves. Moreover, *Aaubp14* deletion significantly suppressed ACT biosynthesis gene expression and blocked ACT production. Comparative proteomics showed Aaubp14 regulates ACT biosynthesis by modulating protein ubiquitination in metabolic pathways and controls pathogenicity via a complex network. Our findings elucidate *Aaubp* gene function in development and pathogenicity, particularly the Aaubp14-mediated regulation mechanism, providing insights into ubiquitination-mediated pathogenicity in phytopathogenic fungi.

## 1. Introduction

The *Alternaria alternata* tangerine pathotype causes Alternaria brown spot, a significant disease affecting susceptible tangerine cultivars (e.g., Dancy, Sunburst, Ponkan, and Ougan), and their hybrids, including tangelos (e.g., Minneola, Murcott, Orlando, and Nova, Lee) and tangors (e.g., Gonggan, Ehime, and Amakusa). The pathogen infects young fruits, leaves, and twigs, causing defoliation, twig dieback, and fruit drop. Infected fruits develop lesions ranging from small spots to large pock marks on the peel, severely reducing their marketability [1,2,3]. The tangerine pathotype of *A. alternata* overwinters in lesions on both attached and fallen mature leaves and shoots. Conidia form on these lesions and are dispersed by air currents when triggered by rainfall under favorable humidity conditions [2,4,5].

The host-specific toxin *Alternaria citri* toxin (ACT) is essential for the pathogenesis of *A. alternata* tangerine pathotype [5,6,7]. ACT, a low-molecular-weight secondary metabolite, comprises 9,10-epoxy-8-hydroxy-9-methyl-decatrienoic acid (EDA), valine, and polyketide compounds. It exhibits potent toxicity toward susceptible citrus cultivars at concentrations as low as 2 × 10^−8^ M [7]. The genes responsible for ACT biosynthesis are clustered on a conditionally dispensable chromosome, containing 22~25 genes that encode hydrolases, oxidoreductases, and transcription factors essential for ACT synthesis [6,8,9,10]. The disruption of the key genes *ACTT1*, *ACTT2*, *ACTT3*, *ACTT5*, *ACTT6*, *ACTTS1*, *ACTTS2*, *ACTTS3*, *ACTTS4*, or *ACTTR* impairs ACT biosynthesis and reduces or even eliminates pathogenicity [5,6,8,11,12,13,14,15,16], demonstrating the essential role of ACT in infection. However, the regulatory network governing ACT biosynthesis remains to be elucidated.

Recent studies have identified multiple pathogenicity-associated genes in the *A. alternata* tangerine pathotype. Critical components include the MAPK signaling pathway proteins (AaFUS3, AaSLT2, and AaHOG1), two-component signal transduction system elements (AaSKN7 and AaSSK1), and calcium signaling pathway factors (AaPLC1 and AaCAL1) [17,18,19,20,21,22]. Additionally, the deletion of transcription factors *AaTfb5*, *AaAreA*, *AaLreA*, *AaLreB*, *AaNsdD*, *AaStuA*, or *AaPacC* significantly impairs virulence [15,23,24,25]. Mutants lacking the non-ribosomal peptide synthetase gene *AaNPS6*, peroxisome-related genes *AaPex3* and *AaPex6*, or the autophagy-related gene *AaAtg8* show markedly reduced pathogenicity [26,27,28,29]. These findings indicated a complex regulatory network in the pathogenicity of the *A. alternata* tangerine pathotype.

Post-translational modifications (PTMs) play crucial regulatory roles in phytopathogenic fungi and have attracted significant research interest. Ubiquitination involves the covalent attachment of ubiquitin, a 76-amino-acid protein, to substrate proteins, thereby modulating their function, localization, and stability [30,31,32,33]. Deubiquitinating enzymes (DUBs) reverse ubiquitination by cleaving ubiquitin–substrate bonds [34]. DUBs perform essential regulatory functions in the ubiquitin system by processing ubiquitin precursors into mature monomers, maintaining free ubiquitin pool homeostasis through chain disassembly, preventing protein degradation via deubiquitylation, and modulating signaling pathways [35]. DUBs are conserved across eukaryotes and comprise six subfamilies: ubiquitin-specific proteases (UBP/USPs), ubiquitin C-terminal hydrolases (UCHs), ovarian tumor proteases (OUTs), Machado-Josephin domain containing proteases (MJDs), JAB1/MPN/MOV34 metalloproteases (JAMMs), and motif interacting with Ub-containing novel DUB family (MINDY). The UBP/USP subfamily represents the largest and most diverse group [34,36].

In plant pathogenic fungi, UBPs regulate hyphal growth, stress responses, pathogenicity, and other processes with both functional redundancy and specificity [37]. Studies in *Magnaporthe oryzae* revealed that *Moubp1*, *Moubp3*, *Moubp4*, *Moubp6*, *Moubp8*, *Moubp12*, and *Moubp14* are essential for colony growth, sporulation, and pathogenicity [38]. Moubp3 modulates Ras-mediated signaling by deubiquitinating Smo1, affecting cAMP and Pmk1-MAPK pathways while suppressing target of rapamycin (TOR) signaling, thus influencing ribosomal autophagy and infection [39]. Moubp4 likely functions in endosomes through interaction with MoBro1, while Moubp8 deubiquitinates histone H2B; both Δ*Moubp4* and Δ*Moubp8* showed reduced virulence [40,41]. In *Fusarium graminearum*, the deletion of *Fgubp3*, *Fgubp14*, or *Fgubp15* leads to pathogenicity defects. *Fgubp14* regulates histone acetyltransferase *Gcn5* expression, affecting H3K18 acetylation levels and deoxynivalenol (DON) toxin synthesis, while Fgubp15 targets Atg8, Pmk1, and Tri4 through deubiquitylation, impacting autophagy, infection structure formation, and toxin production [42]. While these studies demonstrate the diverse functions of UBPs in fungal pathogens, many of their specific regulatory mechanisms await identification.

Despite extensive research on the molecular mechanisms of *A. alternata* pathogenicity, epigenetic studies remain limited, particularly regarding ubiquitination mechanisms. Here, we report the first comprehensive functional analysis of 13 ubiquitin-specific proteases (UBPs) in the tangerine pathotype of *A. alternata*. This study systematically characterized the roles of UBPs in *A. alternata* growth, development, and virulence, with particular focus on elucidating the molecular mechanisms underlying UBP-mediated regulation of ACT biosynthesis and pathogenicity.

## 2. Materials and Methods

### 2.1. Fungal Strains and Culture Conditions

The wild-type *A. alternata* tangerine pathotype strain Z7, with a published chromosome-level genome, was isolated from infected *Citrus reticulata* Blanco cv. Ougan leaves in Wenzhou, Zhejiang, China [3,8]. Z7 and derivative mutants were maintained on Potato Dextrose Agar (PDA) plates at 26 °C and stored in 30% glycerol at −80 °C. For liquid culture, strains were grown in Potato Dextrose Broth (PDB) at 26 °C with shaking at 150 rpm for 2~3 days. Complete medium (CM) and minimal medium (MM) were used for carbon source utilization studies [43,44]. V8 medium was used for conidiation assays [44].

### 2.2. DNA and RNA Extraction, Gene Cloning, and Sequence Analysis

Genomic DNA was extracted using the CTAB method, and total RNA was isolated using RNA Isolator Total RNA Extraction Reagent (R401-01; Vazyme, Nanjing, China) as described previously [45]. PCR primers were designed using NCBI Primer-BLAST (https://www.ncbi.nlm.nih.gov/tools/primer-blast//, accessed on 26 June 2025) and are listed in Supporting Materials Table S1. PCR reactions were performed using a Bio-Rad T100 Thermal Cycler following the manufacturer’s instructions. For phylogenetic analysis, multiple protein sequence alignments were performed using MAFFT (v7.313) and trimmed with trimAl (v1.2). An unrooted maximum likelihood phylogenetic tree was constructed using IQ-TREE (v1.6.8) with 1000 ultrafast bootstrap replicates under the optimal LG + R5 model determined by ModelFinder.

### 2.3. Generate Gene Deletion Mutants and Complementation Strains

Gene deletion mutants were generated using split-marker homologous recombination [46]. The 5’- and 3’- flanking sequences (~1.0 kb) of each target gene were amplified and fused with a hygromycin resistance cassette. The fusion products were transformed into wild-type Z7 protoplasts using polyethylene glycol-mediated transformation [23]. Transformants were selected on plates with hygromycin B at 100 μg/mL (10843555001; Roche, Basel, Switzerland) by three rounds of hyphal tip isolation. For complementation, the full-length gene with its 1.5 kb upstream and 0.5 kb downstream terminators were amplified and cloned into p1300-NEO using ClonExpress MultiS One Step Cloning Kit (C113-01; Vazyme, Nanjing, China). The construct was transformed into corresponding deletion mutants, with transformants selected on plates with neomycin G418 at 100 μg/mL (G274348; Aladdin, Shanghai, China). For epitope tagging, the TrpC promoter, coding sequences of FBP1 or PCK1, 3 × FLAG tag, and TrpC terminator were amplified separately, fused, and cloned into p1300-NEO. The plasmids were transformed into Z7 or Δ*Aaubp14*. All transformants were verified by PCR. Primer sequences are listed in Table S1.

### 2.4. Morphological Features Analysis

For colony morphology analysis, fresh mycelial plugs were inoculated onto 6 cm diameter PDA plates and incubated at 26 °C. Colony diameters were measured at 24 and 48 h post-inoculation (hpi). Daily radial growth rates were calculated using the formula: Growth rate (mm/day) = (diameter at 48 hpi − diameter at 24 hpi)/2 [47]. Hyphal coloration was evaluated by comparing pigment deposition in colonies grown from mycelial plugs on PDA medium for 7 days. For conidiation analysis, strains were grown on V8 medium at 26 °C for 15 days. Conidia were harvested and quantified using a hemocytometer. For germination assays, conidial suspensions with a concentration of 1 × 10^5^ conidia/mL were prepared, and 20 μL aliquots were spotted onto sterile glass slides. After a 12 h incubation at 26 °C in darkness, germination rates were determined by counting 100 randomly selected conidia per replicate. Conidial morphology was analyzed by measuring the length and width of randomly selected conidia per plate using ImageJ v1.54d (National Institutes of Health, Bethesda, MD, USA); transverse and longitudinal septa were counted for each conidium. Microscopic observations were performed using a Nikon Eclipse 80i microscope, and images were captured with NIS-Elements F3.0 software (Nikon, Tokyo, Japan). All experiments above were performed in triplicate.

### 2.5. Pathogenicity Assay

Pathogenicity was assessed on young leaves of *C. reticulata* Blanco cv. Hongjv (hereafter referred to as Hongjv). Young leaves were collected from new shoots approximately three weeks after emergence. Mycelial plugs (3 mm diameter) from 2-day-old colony edges were placed on detached leaves. Inoculated leaves were incubated at 26 °C under almost 100% humidity. Disease symptoms were evaluated at 3 days post-inoculation (dpi), and lesion areas were measured using ImageJ v1.54d (National Institutes of Health, Bethesda, MD, USA). The assay had three biological replicates.

### 2.6. Toxicity Test and UHPLC-MS/MS Analysis of ACT-Toxin

ACT extraction from culture filtrates followed the previously described methods [7,12,23]. Briefly, six fresh mycelial plugs (5 mm diameter) per strain were cultured in Modified Richards’ solution at 26 °C for 24 days. Culture filtrates were incubated with 10 mL of Amberlite XAD-2 resin for 2 h, eluted with methanol, extracted with ethyl acetate, and concentrated to 1 mL to obtain crude ACT. Toxicity was assessed by applying 10 μL of 10-fold-diluted crude ACT to wounded sites on young Hongjv leaves. Then, treated leaves were incubated at 26 °C under 100% humidity.

UHPLC-MS/MS analysis was performed using a Vanquish UHPLC system coupled to either an Orbitrap Q Exactive^TM^ HF or HF-X mass spectrometer (Thermo Fisher, Rheinfelden, Germany) at Novogene Co., Ltd. (Beijing, China). An equal volume of samples was separated on a Hypersil Gold column (100 × 2.1 mm, 1.9 μm) using a 12 min linear gradient at 0.2 mL/min. The eluents for the positive and negative polarity modes were 0.1% formic acid in water (eluent A) and methanol (eluent B). The solvent gradient was set as follows: 2%B, 1.5 min; 2~85%B, 3 min; 85~100%B, 10 min; 100~2%B, 10.1 min; 2%B, 12 min.

### 2.7. Quantitative Real-Time PCR

Gene expression was analyzed in *A. alternata* tangerine pathotype mycelium harvested at 0, 12, 24, 36, and 48 hpi from infected Hongjv leaves. Total RNA was extracted and reverse transcribed using HiScript III 1st Strand cDNA Synthesis Kit (+gDNA wiper) (R312-01; Vazyme, Nanjing, China). qRT-PCR was performed using a BioRad CFX96 Real-Time PCR System with ChamQ Universal SYBR qPCR Master Mix (Q711-02; Vazyme Nanjing, China) following the manufacturer’s instructions. The actin gene *Actin* (NCBI: KP341672) served as a reference. Relative expression was calculated using the 2^−ΔΔCT^ method. Each analysis included three biological replicates with three technical replicates per time point. Primer sequences are listed in Table S1.

### 2.8. Carbon Sources Utilization Test

Carbon source utilization was assessed by inoculating mycelial plugs (5 mm diameter) from 2-day-old colony edges onto MM supplemented with 2% glucose, 2% glycerol, 2% ethanol, or 5 mM sodium acetate as sole carbon source, respectively. CM served as a positive control. Plates were incubated at 26 °C for 3 days. Colony diameter was measured to calculate relative growth inhibition [48,49]. Colony morphology was documented. The experiments included three biological replicates with three technical replicates each.

### 2.9. Protein Extraction and Western Blotting

Protein extraction and Western blot analysis were performed as described previously [50]. Briefly, fresh mycelium was harvested, ground in liquid nitrogen, and resuspended in RIPA buffer (P0045; Beyotime, Shanghai, China) containing a 1% protease inhibitor cocktail for fungal and yeast extracts (P1020; Beyotime, Shanghai, China). Samples were incubated on ice for 30 min with a vortex every 10 min, then centrifuged at 12,000 rpm for 20 min at 4 °C. Supernatants were mixed with 5 × SDS-PAGE Sample Loading Buffer (BL502B; Biosharp, Anhui, China), boiled for 10 min, and separated on 4~20% gradient SDS-PAGE gels (TSP024-15; Tsingke, Beijing, China). Anti-GAPDH (R1208-3; Huaan, Hangzhou, China), Anti-ubiquitin (ER31212; Huaan, Hangzhou, China), and Anti-FLAG (14793; Cell Signaling Technology, Danvers, Massachusetts, USA) were used as primary antibodies. HRP-conjugated goat anti-rabbit IgG (HA1001; Huaan, Hangzhou, China) served as a secondary antibody. Signals were detected using BeyoECL Plus Kit (P0018S; Beyotime, Shanghai, China) according to the manufacturer’s instructions.

### 2.10. LC–MS/MS Analysis of Ubiquitylated Peptides

Ubiquitylated peptides from the WT strain Z7 and the Δ*Aaubp14* mutant were analyzed using LC-MS/MS. Mycelium was harvested at 36 hpi from Hongjv leaves. Total protein was extracted, quantified using the BCA assay, and digested with trypsin. Peptides were dissolved in NETN buffer (100 mM NaCl, 1 mM EDTA, 50 mM Tris-HCl, 0.5% NP-40, pH 8.0) and immunoprecipitated using anti-ubiquitin antibody beads (PTM-1104; PTM Bio, Hangzhou, China) overnight at 4 °C with gentle shaking. Beads were washed four times with NETN buffer and twice with deionized water. Bound peptides were eluted with 0.1% trifluoroacetic acid, vacuum-dried, and desalted using C18 ZipTips (ZTC18S960; Millipore, Darmstadt, Germany). Mass spectrometry analysis was performed at PTM Bio (Hangzhou, China). The resulting MS data were analyzed using DIA-NN (v1.8) against the SwissProt database with a reverse decoy database. The enzyme digestion was set to Trypsin/P, and the maximum missed cleavages was allowed to be two. Variable modification was set to ubiquitination on lysine, and fixed modifications were set as carbamidomethylation on cysteine. The false discovery rate (FDR) for precursor identification was set to 1%.

### 2.11. Bioinformatics Analysis

Gene Ontology (GO) annotation was performed using eggnog-mapper based on the EggNOG database. Proteins were classified according to cellular components, molecular functions, and biological processes [51]. The Kyoto Encyclopedia of Genes and Genomes (KEGG) pathway annotation is based on the KEGG database with BLAST sequence comparison [52]. Protein domains were annotated using the Pfam database and the PfamScan tool. Subcellular localization was predicted using PSORTb. The motif characteristics analysis of the ubiquitinated sites was performed using Motif-X, examining 10 amino acids upstream and downstream of modification sites [53]. Protein–protein interaction networks were constructed using the STRING database for *A. alternata* tangerine pathotype DEPs from proteomic analysis using a combined score threshold of 400 [54]. Based on the pathogenicity-related proteins listed in Table S2, all interaction networks with pathogenicity-related proteins as hub genes were selected and visualized using Cytoscape (3.10.3) [55].

### 2.12. Statistical Analysis

Statistical analyses were conducted using IBM^®^ SPSS Statistics software (V26.0.0.0) to assess differences between experimental groups. One-way ANOVA with Tukey’s test was performed for multiple comparisons, with significant differences at *p* < 0.05 indicated by different letters. For pathogenicity assays comparing lesion areas between wild-type strain Z7 and gene knockout mutants, Student’s *t*-test was used, with significance levels denoted as ** (*p* < 0.01) and *** (*p* < 0.001).

## 3. Results

### 3.1. Characterization of UBPs in the Tangerine Pathotype of A. alternata

**Figure 1:**
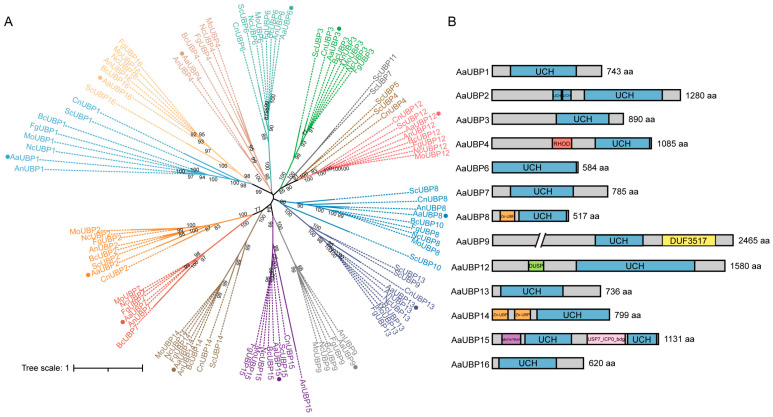
Phylogenetic analysis and domain architecture of the AaUBPs. (**A**) Maximum-likelihood phylogenetic tree of UBPs and their fungal orthologues from *Saccharomyces cerevisiae* (Sc), *Cryptococcus neoformans* (Cn), *Neurospora crassa* (Nc), *Aspergillus nidulans* (An), *Magnaporthe oryzae* (Mo), *Fusarium graminearum* (Fg), *Botrytis cinerea* (Bc), and *Alternaria alternata* tangerine pathotype (Aa, with the mark of “·”). (**B**) Domain organization of AaUBPs. UCH, ubiquitin carboxyl-terminal hydrolase; RHOD, Rhodanese-like domain; zf-UBP, Zn-finger in ubiquitin hydrolases and other proteins; DUSP, domain present in ubiquitin-specific protease; MATH, meprin and TRAF homology; USP7_ICP0_bdg, ICP0-binding domain of ubiquitin-specific protease 7.

*Saccharomyces cerevisiae* contains 16 identified UBPs [37,56]. Using *S. cerevisiae* UBP protein sequences as queries, we identified 13 homologous proteins in the *A. alternata* tangerine pathotype strain Z7 genome (GenBank: GCA_014751505.1). These proteins were designated as AaUBP1~16, with AaUBP5, AaUBP10, and AaUBP11 absent from the genome. To analyze the evolutionary relationships among fungal UBPs, we performed multiple sequence alignment of UBP proteins from eight species: *S. cerevisiae*, *Cryptococcus neoformans*, *Neurospora crassa*, *Aspergillus nidulans*, *M. oryzae*, *F. graminearum*, *Botrytis cinerea*, and the *A. alternata* tangerine pathotype. Maximum likelihood phylogenetic analysis with IQ-TREE revealed that AaUBPs clustered with their respective homologs from other species (Figure 1A), suggesting functional conservation across fungi. InterPro domain analysis (https://www.ebi.ac.uk/interpro/search) revealed that all 13 AaUBPs contain the characteristic UCH domain, which is essential for recognizing and cleaving C-terminal glycine bonds in ubiquitin during precursor processing and protein deubiquitylation [34]. Several UBPs possessed additional functional domains: RHOD (AaUBP4), zf-UBP (AaUBP8 and AaUBP14), DUSP (AaUBP7, AaUBP9, and AaUBP12), DUF3517 (AaUBP9), and both MATH and USP7_ICP0_bdg (AaUBP15) (Figure 1B).

### 3.2. Expression Dynamics of Aaubp Genes During Vegetative Growth and Infection

**Figure 2:**
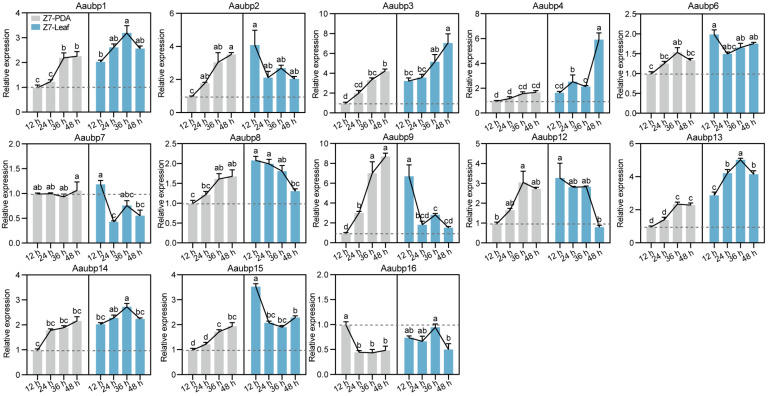
Comparative expression dynamics of *Aaubp* genes during PDA cultivation and leaf infection. Mycelium of wild-type strain Z7 was cultured in PDA medium or inoculated onto Hongjv leaves. Data are presented as means ± SE. Different letters indicate significant differences by Tukey’s test at *p* < 0.05.

The expression patterns of *Aaubp* genes were analyzed by parallel inoculation of wild-type Z7 mycelium onto PDA medium and Hongjv leaves, representing nutritional and infection phases, respectively. Expression levels of 13 *Aaubp* genes were quantified by qRT-PCR at 12, 24, 36, and 48 hpi. Temporal analysis revealed a gradual upregulation of all *Aaubp* genes except *Aaubp16* under PDA culture conditions. Leaf inoculation significantly induced most *Aaubp* genes relative to PDA conditions, though with distinct temporal expression patterns (Figure 2). Specifically, *Aaubp1*, *Aaubp3*, *Aaubp4*, *Aaubp6*, *Aaubp13*, *Aaubp14*, and *Aaubp15* exhibited significantly higher expression levels at all time points (12, 24, 36, and 48 hpi) compared to those of PDA conditions. Among these, *Aaubp3* and *Aaubp4* showed progressive upregulation throughout infection, while *Aaubp1*, *Aaubp13*, and *Aaubp14* peaked at 36 hpi (Figure 2). Additionally, *Aaubp2*, *Aaubp6*, *Aaubp7*, *Aaubp8*, *Aaubp9*, *Aaubp12*, and *Aaubp15* displayed an expression pattern that all peaked at 12 hpi (Figure 2). These results indicate that most *Aaubp* genes are upregulated in response to host infection, suggesting their potentially important roles in pathogenesis.

### 3.3. Characterization of Vegetative Growth of the ΔAaubp Mutants

**Figure 3:**
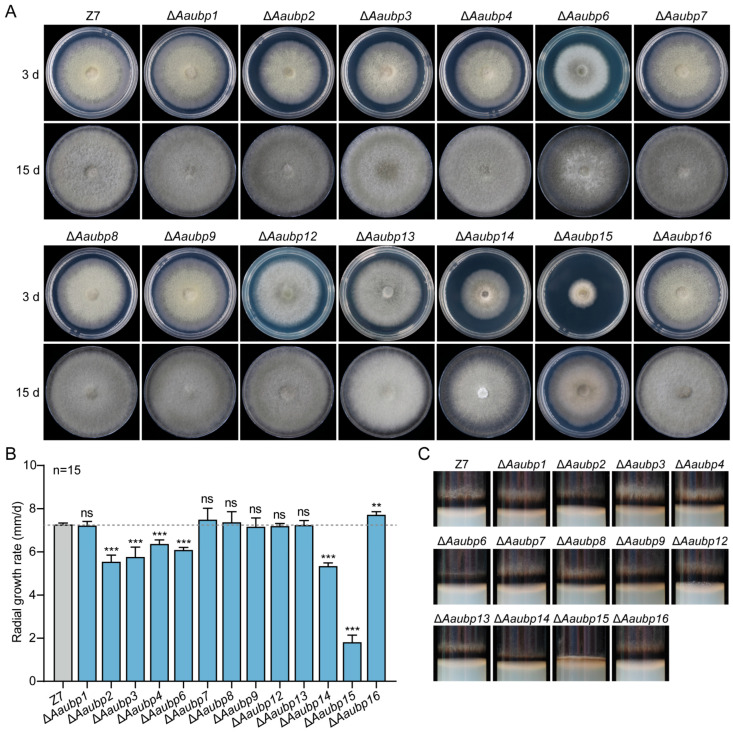
Growth characteristics of Δ*Aaubp* mutants. (**A**) Colony morphology of wild-type strain (Z7) and Δ*Aaubp* mutants grown on 6 cm PDA plates at 26 °C for 3 and 15 days. (**B**) Radial growth rates of colonies. Data are presented as means ± SE. Statistical significance was determined by Student’s *t*-test compared to wild-type Z7 (ns, not significant; ** *p* < 0.01; *** *p* < 0.001). (**C**) Hyphal coloration after 7 days of cultivation on PDA medium.

Single-gene deletion mutants of all 13 *Aaubp* genes were generated in the wild-type Z7 strain via homologous recombination and confirmed by PCR analysis (Figure S1). Some *Aaubp* gene deletion mutants showed altered colony morphology and growth to some degree compared to WT (Figure 3). Deletion of *Aaubp2*, *Aaubp3*, *Aaubp4*, *Aaubp6*, *Aaubp14*, and *Aaubp15* significantly reduced mycelial growth rates, with Δ*Aaubp15* showing the most severe reduction (Figure 3A,B). Conversely, Δ*Aaubp16* exhibited enhanced radial growth (Figure 3A,B). In addition, Δ*Aaubp15* displayed lighter colony coloration relative to Z7 (Figure 3C). These findings demonstrate that *Aaubp* genes differentially regulate fungal vegetative growth.

### 3.4. Characterization of Conidiation and Conidial Morphology of the ΔAaubp Mutants

**Figure 4:**
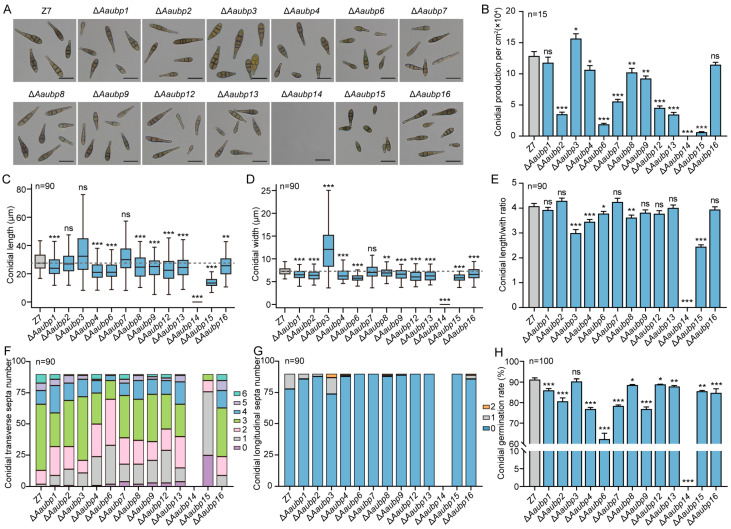
Conidial characteristics of Δ*Aaubp* mutants. (**A**) Microscopic morphology of conidia from wild-type (Z7) and Δ*Aaubp* mutants (scale bar = 20 μm). (**B**) Conidial production after 15 days of growth on V8 medium at 26 °C. (**C**–**G**) Quantification of conidial morphology: (**C**) length, (**D**) width, (**E**) length-to-width ratio, (**F**) number of transverse septa, and (**G**) number of longitudinal septa. (**H**) Conidial germination rate. Scale bar = 20 μm. Data are presented as means ± SE. Statistical significance was determined by Student’s *t*-test compared to wild-type Z7 (ns, not significant; * *p* < 0.05; ** *p* < 0.01; *** *p* < 0.001).

Conidia play a critical role in the disease cycle of citrus Alternaria brown spot. Among the thirteen Δ*Aaubp* mutants, Δ*Aaubp14* exhibited complete loss of sporulation capacity, ten exhibited reduced sporulation, while Δ*Aaubp3* showed enhanced sporulation. Compared to wild-type Z7, severe sporulation defect (>50% reduction) was observed in Δ*Aaubp2*, Δ*Aaubp6*, Δ*Aaubp7*, Δ*Aaubp12*, Δ*Aaubp13*, and Δ*Aaubp15*. The Δ*Aaubp9* mutant showed moderate reduction (25–50%), while Δ*Aaubp4* and Δ*Aaubp8* exhibited mild reduction (<25%) in spore production (Figure 4A,B). In addition, we found that most Aaubp deletions significantly reduced conidial length, with the exception of Δ*Aaubp2*, Δ*Aaubp3*, and Δ*Aaubp7* (Figure 4C). Most Δ*Aaubp* mutants also displayed reduced conidial width, whereas Δ*Aaubp3* deletion resulted in increased width and no significant changes in Δ*Aaubp7* (Figure 4D). Analysis of conidial length-to-width ratios revealed reductions in Δ*Aaubp3*, Δ*Aaubp4*, Δ*Aaubp6*, Δ*Aaubp8*, and Δ*Aaubp15* (Figure 4E). Moreover, several mutants exhibited reduced transverse septation, with Δ*Aaubp6* and Δ*Aaubp15* showing the most pronounced decrease (Figure 4F), and longitudinal septation was absent in Δ*Aaubp6*, Δ*Aaubp7*, Δ*Aaubp12*, Δ*Aaubp13*, and Δ*Aaubp15* conidia after 15 days of incubation on V8 medium (Figure 4G). These findings suggest that *Aaubp* genes are essential for proper conidial morphology. Conidial germination was impaired in all *Aaubp* mutants except Δ*Aaubp3*. While Z7 showed 91.22% germination after 12 h incubation in 2% glucose at 26 °C, germination rates of Δ*Aaubp4*, Δ*Aaubp6*, Δ*Aaubp7*, and Δ*Aaubp9* fell below 80%, with Δ*Aaubp6* showing the lowest germination rate of 62.33% (Figure 4H), demonstrating the importance of *Aaubp* genes in conidial germination.

### 3.5. The Aaubp Genes Are Involved in Pathogenicity

**Figure 5:**
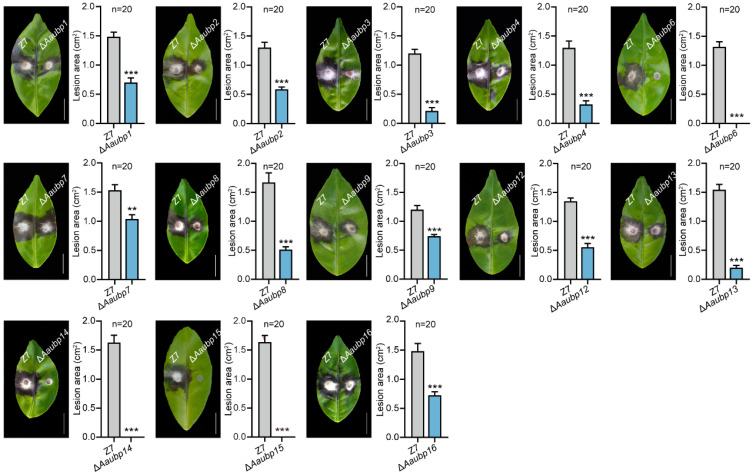
Pathogenicity analysis of Δ*Aaubp* mutants. Mycelial plugs (3-mm diameter) were inoculated onto Hongjv citrus leaves and incubated at 26 °C with 100% humidity. Disease symptoms were evaluated at 72 hpi. Lesion areas were measured using ImageJ software (1.54d). Scale bar = 1 cm. Data are presented as means ± SE. Statistical significance was determined by Student’s *t*-test compared to wild-type Z7 (** *p* < 0.01; *** *p* < 0.001).

To investigate the involvement of *Aaubp* genes in pathogenicity, we inoculated mycelial plugs of *Aaubp* deletion mutants on Hongjv leaves. Notably, the Δ*Aaubp6*, Δ*Aaubp14*, and Δ*Aaubp15* mutants showed complete loss of pathogenicity, and the remaining ten *Aaubp* gene deletion mutants developed significantly smaller lesions compared to the wild-type strain Z7 (Figure 5). These results establish UBP family proteins as critical virulence regulators in the *A. alternata* tangerine pathotype, with *Aaubp6*, *Aaubp14*, and *Aaubp15* being essential for pathogenicity.

### 3.6. Deletion of Aaubp14 Disrupts the Biosynthesis of ACT-Toxin

**Figure 6:**
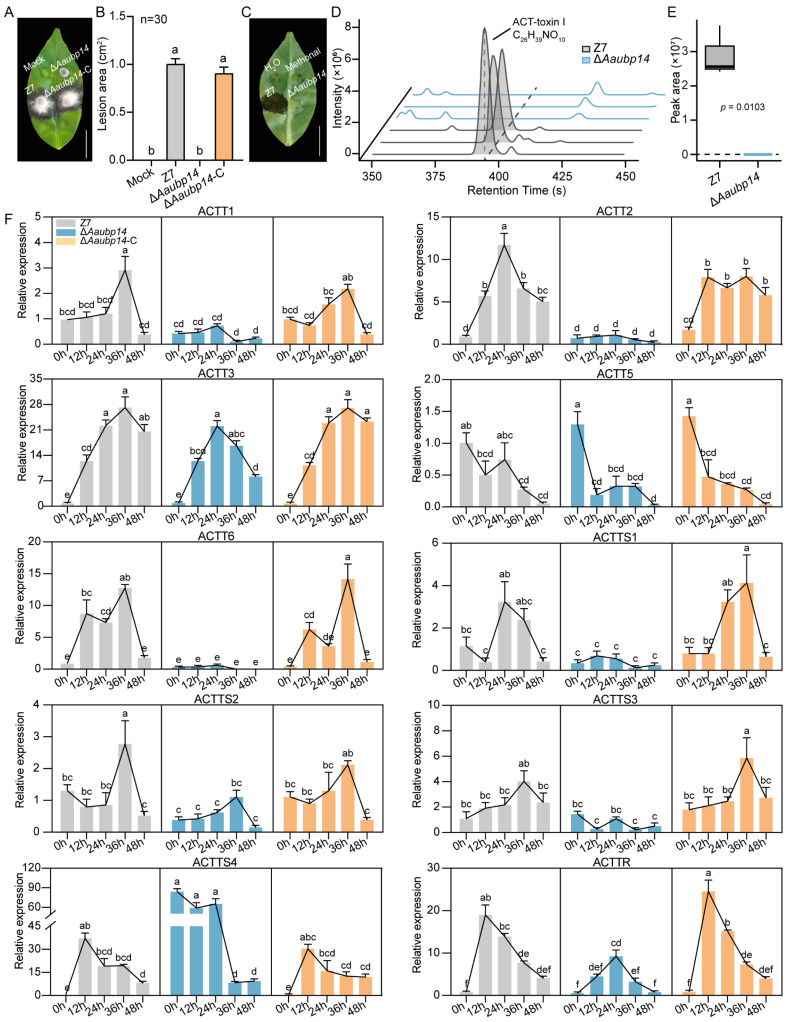
Aaubp14 regulates ACT-toxin biosynthesis. (**A**) Pathogenicity assay of wild-type Z7, Δ*Aaubp14* mutant, and complemented strain Δ*Aaubp14*-C on Hongjv leaves. Mycelial plugs (3 mm in diameter) were inoculated and incubated at 26 °C for 72 h. (**B**) Quantification of lesion areas at 72 hpi using ImageJ software (1.54d). (**C**) Virulence assay on Hongjv leaves using crude toxin extracts from Z7 and Δ*Aaubp14*, assessed at 36 hpi. (**D**) UHPLC-MS/MS detection of ACT-toxin I in Z7 and Δ*Aaubp14*. (**E**) Quantitative comparison of ACT-toxin I levels between Z7 and Δ*Aaubp14* strains by UHPLC-MS/MS. Peak areas were normalized based on the dry weight ratio of Z7 to Δ*Aaubp14* mycelial biomass. (**F**) Expression analysis of *ACTT* genes in Z7, Δ*Aaubp14*, and Δ*Aaubp14*-C strains after Hongjv leaf inoculation by qRT-PCR. Different letters indicate significant differences by Tukey’s test at *p* < 0.05.

The Δ*Aaubp14* mutant’s inability to produce spores and loss of pathogenicity strongly suggest that Aaubp14 is crucial for sporulation and virulence. To explore the regulatory mechanism of Aaubp14, we generated the complemented strain Δ*Aaubp14*-C and conducted the pathogenicity using leaf inoculation. While the Δ*Aaubp14* mutant exhibited complete loss of pathogenicity, the complemented strain Δ*Aaubp14*-C restored virulence to wild-type levels (Figure 6A,B). ACT is the primary virulence determinant of *A. alternata* tangerine pathotype. Two types of ACT, I and II, have been reported. ACT-toxin I is more abundant and highly toxic to both citrus and pear, while ACT-toxin II is not toxic to citrus [7]. Crude ACT was extracted following established protocols [13,57] and applied to wounded immature Hongjv leaves [7]. After incubation at 26 °C under humid conditions, crude toxin from wild-type Z7 induced lesions at inoculation sites, while crude toxin of Δ*Aaubp14* caused no visible lesions (Figure 6C). UHPLC-MS/MS analysis was conducted to identify the components of crude toxin from Z7 and Δ*Aaubp14*. The results revealed the absorption peaks of ACT-toxin I in Z7, similar to previous reports [7], whereas these peaks were absent in the Δ*Aaubp14* mutant (Figure 6D,E). Additionally, ACT-toxin II absorption peaks were detected in Z7 but showed a significant reduction in the Δ*Aaubp14* mutant (Figure S2).

ACT biosynthesis is coordinated by multiple genes located within the *ACTT* gene cluster. Disruption of any *ACTT* gene significantly abolishes or reduces ACT production and pathogenicity, indicating their essential role in virulence [5,6,8,11,12,13,14,15,16]. We analyzed *ACTT* gene expression in mycelia that were inoculated on Hongjv leaves at 0, 12, 24, 36, and 48 hpi. As shown in Figure 6F, leaf inoculation induced expression of *ACTT1*, *ACTT2*, *ACTT3*, *ACTT6*, *ACTTS1*, *ACTTS2*, *ACTTS3*, *ACTTS4*, and *ACTTR* in both Z7 and complemented strains. Among these genes, *ACTTR* showed the highest expression level at 12 hpi, while *ACTT2* and *ACTTS1* peaked at 24 hpi, and *ACTT1*, *ACTT3*, *ACTT6*, *ACTTS2*, and *ACTTS3* peaked at 36 hpi. However, *Aaubp14* deletion significantly suppressed the expression of these genes upon leaf exposure compared to Z7 and Δ*Aaubp14*-C (Figure 6F). Of particular note, *ACTTS4* expression was significantly elevated in the Δ*Aaubp14* mutant at 0, 12, and 24 hpi compared to Z7 and Δ*Aaubp14*-C, suggesting that *Aaubp14* negatively regulates *ACTTS4*. The expression of *ACTT5* was relatively stable across all strains and exhibited minimal inoculation response (Figure 6F). Collectively, these results demonstrate that Aaubp14 functions as a critical regulator of ACT biosynthesis by modulating transcription within the *ACTT* gene cluster.

### 3.7. Quantitative Proteomics Analysis of Hyper-Ubiquitinated Proteins in ΔAaubp14

**Figure 7:**
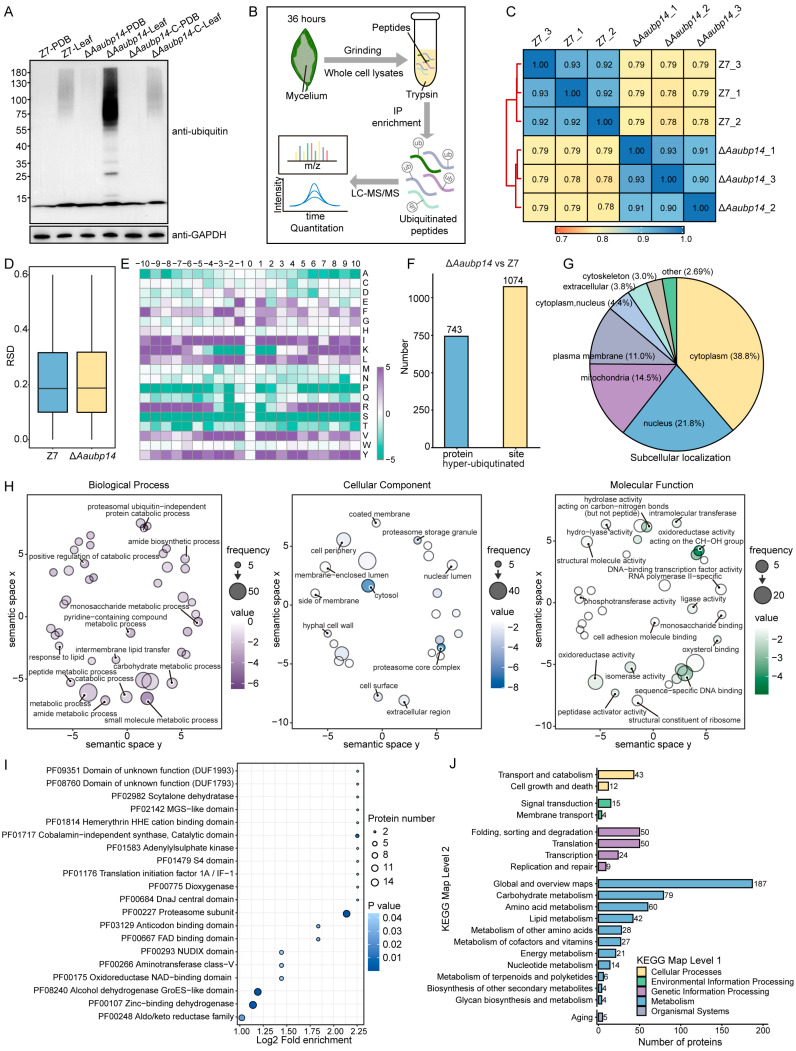
Global ubiquitinome analysis of Δ*Aaubp14* during infection. (**A**) Western blot analysis of total protein ubiquitination in wild-type and Δ*Aaubp14* mutant. (**B**) Workflow of quantitative ubiquitinome analysis. Mycelium from wild-type and Δ*Aaubp14* strains was inoculated onto Hongjv leaves (26 °C, 36 h), followed by protein extraction, trypsin digestion, ubiquitin peptide enrichment, and LC-MS/MS analysis. (**C**) Pearson correlation analysis of biological replicates shown as a heatmap. Darker blue shades reflect stronger correlations approaching 1. (**D**) Relative standard deviation (RSD) distribution of quantified proteins. Smaller RSD values indicate better quantitative reproducibility. (**E**) Amino acid frequency analysis of ubiquitination sites. (**F**) Distribution of hyper-ubiquitinated proteins and modification sites identified in Δ*Aaubp14*. (**G**) Subcellular distribution of hyper-ubiquitinated proteins. (**H**) GO enrichment analysis of biological processes. (**I**) Enriched protein domains among hyper-ubiquitinated proteins. (**J**) KEGG pathway enrichment analysis of hyper-ubiquitinated proteins.

Since Aaubp14 is a deubiquitinating enzyme, we analyzed ubiquitination level in mycelia harvested at 36 hpi from both Hongjv leaves and PDB medium of Z7, Δ*Aaubp14*, and the complemented strain Δ*Aaubp14*-C. Western blot analysis revealed stronger ubiquitination signals in mycelia from Hongjv leaves compared to the PDB medium (Figure 7A), suggesting enhanced protein ubiquitination during infection. The Δ*Aaubp14* mutant showed significantly elevated ubiquitination levels in both conditions compared to Z7 and Δ*Aaubp14*-C (Figure 7A), confirming Aaubp14’s role in regulating ubiquitination during pathogenesis.

To elucidate how Aaubp14-mediated deubiquitylation regulates ACT synthesis and pathogenicity, we performed quantitative ubiquitinome analysis of Z7 and Δ*Aaubp14* at 36 hpi on Hongjv leaves (Figure 7B). Both strains showed high intra-group correlation but significant inter-group differences in Pearson’s correlation analysis (Figure 7C). Comparable relative standard deviation distributions confirmed data reliability (Figure 7D). Motif-X analysis of ubiquitination sites revealed enrichment patterns in the surrounding amino acid sequences (−10 to +10 positions). Isoleucine, leucine, valine, and tyrosine were enriched at most positions, while lysine showed enrichment at positions −10 to −6 and 6 to 10, and arginine at positions −10 to −4 and 4 to 10 (Figure 7E). The Δ*Aaubp14* mutant exhibited 1074 significantly hyper-ubiquitinated sites (fold change ≥ 1.5, *p* ≤ 0.05) corresponding to 743 proteins compared to Z7 (Figure 7F). Subcellular localization analysis revealed that most hyper-ubiquitinated proteins resided in the cytoplasm (38.8%), nucleus (21.8%), mitochondria (14.5%), and cell membrane (11.0%), with smaller fractions in the extracellular space and cytoskeleton (Figure 7G). The predominant cytoplasmic and nuclear localization (60.6%) suggests that *Aaubp14* deletion primarily disrupts the ubiquitination balance of proteins in these compartments, potentially affecting gene expression, protein synthesis, and degradation.

### 3.8. Functional Insights into Aaubp14-Mediated Ubiquitinated Proteins

To further investigate the biological functions associated with Aaubp14-mediated deubiquitylation, Gene Ontology (GO) enrichment analysis was performed. Enriched biological processes included small-molecule metabolism, lipid response, and proteasomal ubiquitin-independent protein catabolism. In terms of cellular components, cytosol, proteasome core complex, proteasome storage granule, cell periphery, and cell surface were enriched. Molecular functions showed significant enrichment in oxidoreductase activity, hydrolase activity, and sequence-specific DNA binding (Figure 7H). Domain analysis of hyper-ubiquitinated proteins identified enrichment of proteasome subunit, cobalamin-independent synthase, oxidoreductase NAD-binding domain, alcohol dehydrogenase GroES-like domain, and zinc-binding dehydrogenase domains (Figure 7I). Additionally, KEGG pathway analysis revealed enrichment in multiple metabolic pathways, specifically in carbohydrate, amino acid, and lipid metabolism, as well as terpenoid and polyketide biosynthesis. Signal transduction and transport pathways were also notably enriched (Figure 7J). These results indicate that deletion of *Aaubp14* disrupts ubiquitination patterns, leading to impaired protein degradation, transport, and metabolic processes.

### 3.9. Aaubp14 Regulates the Expression of Virulence-Related Protein

**Figure 8:**
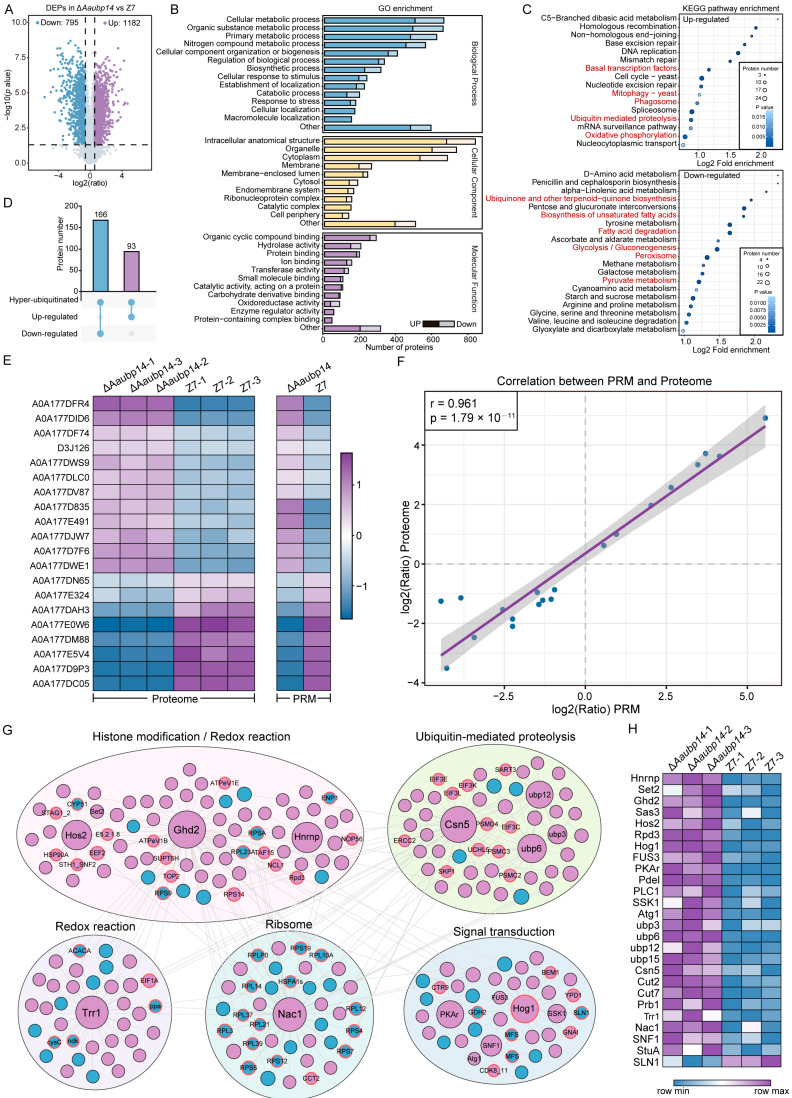
Comparative proteomic analysis reveals Aaubp14-mediated regulation of pathogenicity-related proteins. (**A**) Volcano plot analysis of DEPs between wild-type (Z7) and Δ*Aaubp14* mutant. Blue: downregulated DEPs; purple: upregulated DEPs; gray: non-differentially expressed proteins. (**B**) GO terms of DEPs between Z7 and Δ*Aaubp14*. (**C**) KEGG pathway enrichment analysis of DEPs between Z7 and Δ*Aaubp14*. (**D**) Upset plot showing the overlap between DEPs in Δ*Aaubp14* and Z7 and hyper-ubiquitinated proteins in Δ*Aaubp14*. Blue dots indicate sets participating in each intersection, gray dots represent sets not involved in the specific intersection. Dot size reflects intersection size. (**E**) Heatmap comparing proteomic and SLN results of randomly selected 20 DEPs in Δ*Aaubp14* and Z7. (**F**) Scatter plot depicting the correlation of DEPs abundance ratios between proteomic and PRM analyses. The blue dots represent individual data points of DEPs. The purple regression line and shaded confidence interval highlight the linear relationship. (**G**) Protein–protein interaction network of DEPs related to virulence. Red circles indicate hyper-ubiquitinated in Δ*Aaubp14*. (**H**) Expression profiles of known pathogenicity-related proteins in Z7 and Δ*Aaubp14*.

Furthermore, proteomic analysis of WT Z7 and Δ*Aaubp14* revealed widespread changes in protein expression. In total, 1182 significantly upregulated and 795 downregulated proteins (fold change ≥ 1.5, *p* ≤ 0.05) in Δ*Aaubp14* were identified (Figure 8A). To explore the regulatory network of Aaubp14, we conducted GO analysis to identify the biological processes modulated by its deubiquitylation. Within biological processes, proteins were primarily annotated to functions in cellular metabolism, biosynthesis, and cellular localization. In terms of cellular components, proteins were predominantly found in organelles, cytoplasm, membranes, and ribonucleoprotein complexes. Regarding molecular functions, proteins exhibited notable presence in catalytic activity, protein binding, and ion binding categories (Figure 8B). These indicate that *Aaubp14* deletion affects metabolism, protein transport, and signal transduction.

KEGG pathway analysis revealed upregulation of protein degradation pathways (mitophagy, phagosome formation, ubiquitin-mediated proteolysis), basal transcription factors, and oxidative phosphorylation (Figure 8C), suggesting that Δ*Aaubp14* may activate compensatory degradation pathways to maintain proteostasis. Conversely, downregulated pathways included ubiquinone and terpenoid-quinone biosynthesis, unsaturated fatty acid biosynthesis, fatty acid degradation, peroxisome metabolism, and pyruvate metabolism (Figure 8C). The inhibition of secondary metabolite biosynthesis, particularly fatty acid metabolism, likely restricts ACT precursor production, contributing to reduced pathogenicity.

To further investigate whether the differentially expressed proteins (DEPs) were involved in Aaubp14-mediated ubiquitination, we identified 259 proteins that overlapped between differentially expressed and hyper-ubiquitinated proteins in the Δ*Aaubp14* mutant under infection conditions. Among these, 93 proteins showed both hyper-ubiquitination and significant upregulation, approximately half the number of proteins (166) that exhibited hyper-ubiquitination and significant downregulation (Figure 8D).

We validated the quantitative proteomics data by analyzing 20 randomly selected DEPs from Δ*Aaubp14* using Parallel Reaction Monitoring (PRM). Expression patterns of the selected proteins showed strong concordance between proteomics and PRM analyses (Figure 8E). Correlation analysis revealed a significant positive correlation between proteomics and PRM log2 ratios (r = 0.961, *p* = 1.79 × 10^−11^) (Figure 8F). These results validate the reliability of our proteomics data and the observed differential protein expression patterns.

Then, we constructed the protein–protein interaction (PPI) network of DEPs in Δ*Aaubp14*, with emphasis on pathogenicity-related proteins. Five major modules were identified, comprising histone modification, ubiquitin-mediated protein degradation, signal transduction, ribosome assembly, and redox reactions. Ghd2, Hos2, Csn5, Hog1, PKAr, SNF1, Nac1, and Trr1 were identified as hub proteins (Figure 8G). Previous studies have shown that disruption of these hub proteins resulted in virulence defects in *A. alternata* tangerine pathotype [18,43,44,58,59,60,61,62]. Upon *Aaubp14* deletion, Hog1 and SLN1 showed both enhanced ubiquitination and altered expression levels (Figure 8G,H), indicating direct regulation of Aaubp14-mediated ubiquitination. In contrast, while many proteins in the modules showed increased ubiquitination levels, the hub proteins (Ghd2, Hos2, Csn5, PKAr, SNF1, Nac1, and Trr1) exhibited significant upregulation only in expression levels, without notable increases in ubiquitination (Figure 8G,H), suggesting that the hub proteins may be regulated by the ubiquitination status of their interacting partners. Furthermore, lots of previously reported pathogenesis-related proteins showed significant differential expression in Δ*Aaubp14* (Figure 8H; Table S2). This implies that Aaubp14 regulates multiple pathogenesis-related proteins at the protein level to participate in diverse pathogenic pathways. These findings indicate that Aaubp14 modulates intracellular protein expression levels through a complex network of direct and indirect ubiquitination-dependent mechanisms.

## 4. Discussion

Ubiquitination, a critical post-translational modification, regulates protein degradation, cell signaling, and stress responses [30,63]. DUBs remove ubiquitin from substrate proteins, thereby maintaining cellular ubiquitin homeostasis and protein function [35]. UBPs, the largest DUB subfamily, play crucial roles in cellular processes of eukaryotes [34,64]. We identified 13 conserved UBPs in *A. alternata* tangerine pathotype and characterized their roles in development and pathogenicity. Through proteomics analysis, we demonstrated that Aaubp14 regulates protein stability, signal transduction, and secondary metabolite biosynthesis via the ubiquitination process, affecting mycelial growth, sporulation, ACT biosynthesis, and pathogenesis.

UBPs exhibit diverse functions. Our study revealed that mutants of different *Aaubp* genes exhibited distinct phenotypic defects, suggesting both functional specialization and redundancy. Similar patterns exist in other fungi. In *S. cerevisiae*, 16 UBP genes regulate growth, stress response, nutrient utilization, energy metabolism, and sexual reproduction [34,65]. In *M. oryzae*, deletion of multiple UBPs affects colony growth and conidiation. Δ*Moubp1*, Δ*Moubp3*, Δ*Moubp4*, Δ*Moubp6*, Δ*Moubp8*, and Δ*Moubp14* mutants showed decreased pathogenicity through reduced appressorium formation and penetration efficiency [38,39,49]. Similarly, in *F. graminearum*, deletion of *Fgubp3*, *Fgubp8*, *Fgubp14*, and *Fgubp15* resulted in growth retardation; *Fgubp3*, *Fgubp4*, and *Fgubp14* deletion impact conidiation and conidial morphology, and Δ*Fgubp3*, Δ*Fgubp14*, and Δ*Fgubp15* displayed reduced pathogenicity [42,66]. In our study, *Aaubp1*, *Aaubp2*, *Aaubp3*, *Aaubp4*, *Aaubp14*, and *Aaubp15* regulated mycelial growth, while most affected conidial production and germination. Notably, Δ*Aaubp14* completely lost conidiation ability. Δ*Aaubp3*, Δ*Aaubp4*, Δ*Aaubp6*, Δ*Aaubp8*, and Δ*Aaubp15* showed altered conidial morphology, with Δ*Aaubp6* and Δ*Aaubp15* exhibiting obviously reduced septation. Importantly, qRT-PCR analysis revealed differential induction patterns among all 13 UBP-encoding genes upon exposure to Hongjv leaves. Pathogenicity assays revealed a hierarchical contribution of UBP genes, as mutants Δ*Aaubp6*, Δ*Aaubp14*, and Δ*Aaubp15* completely lost pathogenicity, whereas other *Aaubp* gene deletion mutants displayed reduced virulence compared to the wild-type. These findings demonstrate the diverse roles of UBPs in *A. alternata*. The various functions of UBPs across phytopathogen species demonstrate their adaptation to specific ecological niches and the evolution of distinct infection mechanisms. The key UBP genes offer promising targets for developing novel disease control strategies.

In this study, the deletion of *Aaubp14* impaired mycelial growth, abolished conidiation, and eliminated pathogenicity on Hongjv leaves, showing its essential role in development and virulence. Previous studies have demonstrated that UBP14 homologs perform diverse functions in different fungi. In *S. cerevisiae*, UBP14 regulates gluconeogenic enzyme degradation and cellular ubiquitination levels [67]. In *Metarhizium robertsii*, Mrubp14 facilitates pathogenesis by regulating appressorium-dependent host penetration [68]. In *M. oryzae*, Moubp14 regulates carbon metabolism by stabilizing key glycolytic and gluconeogenic enzymes MoFBP1 and MoPCK1 through deubiquitylation, influencing appressorium formation and turgor maintenance during infection [49]. In *F. graminearum*, the nuclear-localized Fgubp14 directly interacts with FgGcn5, and its deletion reduces FgGcn5 protein levels, resulting in decreased H3K18 acetylation, impaired DON toxin synthesis, enhanced autophagy, and loss of infection structure formation [66]. These suggest the diverse essential roles and distinct regulatory mechanisms of UBP14 homologs across fungi. However, the specific mechanisms by which Aaubp14 regulates development and pathogenicity remain unknown.

In *S. cerevisiae*, UBP14 uniquely functions in disassembling unanchored ubiquitin chains and maintaining ubiquitin homeostasis [34]. Its deletion results in free ubiquitin chain accumulation and ubiquitin monomer depletion, disrupting cellular ubiquitination and ubiquitin-proteasomal degradation. This suggests that UBP14 may require intermediate enzymes to release ubiquitin chains from substrates rather than acting directly on substrate-conjugated ubiquitin [34,69,70,71]. Our quantitative ubiquitin proteomics revealed 743 hyper-ubiquitinated proteins in the Δ*Aaubp14* mutant during infection, suggesting that Aaubp14 may indirectly regulate protein ubiquitination through modulation of free ubiquitin chain dynamics.

ACT serves as the primary virulence determinant in *A. alternata* tangerine pathotype [5,72]. Our study found that the Δ*Aaubp14* mutant showed significant downregulation of ACT biosynthetic genes. Comparative proteomics revealed that *Aaubp14* deletion perturbed multiple metabolic pathways. Elevated ubiquitination of metabolism-related proteins, particularly those involved in lipid, terpene, and polyketide biosynthesis, likely promotes their degradation or inhibits their function. This metabolic network disruption may impair the production of ACT precursors. Additionally, GO analysis of hyper-ubiquitinated proteins revealed enrichment in oxidoreductase and hydrolase activities. These enzymes, potentially critical for the lipid compound ACT biosynthesis, may be functionally compromised by increased ubiquitination, directly affecting toxin production. Quantitative proteomics also demonstrated reduced expression of fatty acid metabolism and peroxisome-related proteins in the Δ*Aaubp14* mutant. Disrupted fatty acid metabolism may affect ACT precursor and intermediate synthesis. Peroxisomes, which facilitate fatty acid β-oxidation [73], are proposed to participate in ACT-toxin EDA moiety synthesis [5]. Supporting this hypothesis, deletion of peroxisome-related genes *AaPex3*, *Aapex6*, *AaPex13*, and *AaPex14* inhibits ACT production [27,29,74], and in our results, Aapex3 was hyper-ubiquitinated in Δ*Aaubp14*. In addition, UHPLC-MS/MS analysis revealed a significant reduction in five major non-host specialized secondary metabolites, Alternuene (ALT), Aternariol methyl ether (AME), Alternariol (AOH), Tenuazonic acid (TEA), and Tentoxin (TEN), in the Δ*Aaubp14* mutant compared to wild-type Z7 (Figure S2), indicating potential broader impacts on metabolite biosynthesis. Our results suggest that Aaubp14 plays a crucial role in regulating ACT biosynthesis by modulating cellular ubiquitination processes.

DUBs play established roles in transcription factor regulation and gene expression control. In *S. cerevisiae*, Ubp8, a key component of the SAGA (Spt-Ada-Gcn5-acetyltransferase) complex (Ubp8/Sgf11/Sus1/Sgf73), regulates transcription through H2B deubiquitylation and subsequent modulation of H3K4 and H3K36 methylation [75,76]. *M. oryzae* Moubp8 is thought to participate in the MoCreA-mediated Carbon Catabolite Repression (CCR) system through undefined mechanisms [40]. In *Arabidopsis thaliana*, UBP14 stabilizes the ELONGATED HYPO-COTYL5 (HY5) transcription factor through deubiquitylation, promoting photomor-phogenesis [77]. The regulatory role of Aaubp14 in fungal transcription factor-mediated development and infection remains unexplored. Our findings show that Δ*Aaubp14* lacks conidiation capacity. Given that BrlA, AbaA, and WetA function as key transcription factors in ascomycete asexual sporulation [78], the investigation of Aaubp14’s interaction with these factors is warranted. Additionally, we observed increased protein levels but decreased ubiquitination of the APSES transcription factor AaStuA in Δ*Aaubp14*. Since Δ*AaStuA* exhibits defective conidiation, impaired ACT synthesis, and lost pathogenicity [25], Aaubp14 may regulate AaStuA activity through ubiquitination-dependent mechanisms. The precise molecular mechanisms underlying these interactions require further investigation.

Notably, KEGG pathway analysis revealed that significantly upregulated proteins related to mitophagy and phagosome formation were enriched in Δ*Aaubp14* during infection. In *M. oryzae*, Moubp3 regulates ribophagy during nitrogen starvation and rapamycin treatment; the Δ*Moubp3* mutant exhibits constitutive TOR pathway activation, suppressing autophagy and affecting appressorium formation and pathogenicity [39]. In *F. graminearum*, Fgubp14 functions through FgGcn5 histone acetyltransferase deubiquitylation. Deletion of either *FgGcn5* or *Fgubp14* enhances autophagy, indicating their negative regulatory roles [66]. This aligns with elevated levels of autophagy-related proteins, including AaAtg1, in Δ*Aaubp14*, which functions in autophagy formation and infection processes of the *A. alternata* tangerine pathotype [79]. Additionally, the Δ*Aaubp14* mutant showed elevated expression of pathogenicity-related histone modifiers, including histone deacetylase Hos2, methyltransferases Set2 and Hnrnp, and demethylase Ghd2. The deletion of these genes in the *A. alternata* tangerine pathotype impairs pathogenicity, with Δ*AaSet2* and Δ*AaGhd2* mutants showing reduced ACT synthesis [58,59]. In this study, quantitative ubiquitination analysis revealed decreased Hnrnp ubiquitination in Δ*Aaubp14*, suggesting Aaubp14’s role in Hnrnp-mediated regulation by stabilizing protein levels. However, AaHos2, AaSet2, and AaGhd2 showed increased protein levels without ubiquitination changes following *Aaubp14* deletion. We hypothesize that Aaubp14 may regulate transcription through the deubiquitylation of transcriptional repressors or indirectly influence protein modifications through key regulatory factor deubiquitylation.

Furthermore, the Δ*Aaubp14* mutant exhibited elevated levels of deubiquitinating enzymes Aaubp3, Aaubp6, and Aaubp12, and deubiquitinating-like protein AaCsn5, with Ubp6 showing decreased ubiquitination. Studies in *S. cerevisiae* have demonstrated that Ubp6 employs both catalytic and non-catalytic mechanisms to regulate protein degradation. Through its catalytic activity, Ubp6 removes ubiquitin modifications from substrates, while its non-catalytic function delays proteasome-mediated degradation to control substrate degradation patterns. These functions establish Ubp6 as a key regulator of proteasomal activity [80]. In this study, we propose that *Aaubp14* deletion impairs Aaubp6 ubiquitination, potentially compromising its functionality. This may trigger compensatory upregulation of Aaubp6 protein levels to counteract the functional deficits caused by reduced ubiquitination. The impaired degradation of free ubiquitin chains in Δ*Aaubp14* may disrupt ubiquitin–proteasome system function, leading to compensatory upregulation of Aaubp3, Aaubp12, and AaCsn5 to maintain protein homeostasis. These proposed mechanisms require further experimental validation.

Moreover, we constructed protein–protein interaction networks by integrating the DEPs from the Δ*Aaubp14* mutant with known pathogenicity-related proteins. Notably, MAP kinases FUS3 and Hog1, the Hog1 upstream regulator SSK1, PKA regulatory subunit PKAr, sucrose non-fermenting protein kinase complex α catalytic subunit SNF1, and thioredoxin reductase Trr1 were differentially expressed in the Δ*Aaubp14* mutant. Previous studies have established the critical roles of these proteins in *A. alternata* tangerine pathotype pathogenicity [17,18,22,44,60,62]. Collectively, these results indicate that Aaubp14 acts as a key regulator in complex signaling networks, coordinating multiple established virulence factors during pathogenesis.

Likewise, UBP14 regulates carbon source utilization in both *S. cerevisiae* and *M. oryzae* [49,67]. Growth analysis of wild-type Z7, Δ*Aaubp14*, and Δ*Aaubp14*-C strains on single carbon sources (glucose, glycerol, ethanol, or sodium acetate) revealed significant growth inhibition of Δ*Aaubp14* under all conditions, confirming Aaubp14’s role in carbon metabolism (Figure S3A,B). While *M. oryzae* Moubp14 mediates glucose-induced degradation of MoFbp1 and MoPck1 through the ubiquitin-proteasome system [49], *A. alternata* shows distinct regulation. Western blot assays revealed decreased levels of AaFBP1 and AaPCK1 in Δ*Aaubp14* following mycelial transfer from sodium acetate- to glucose-containing medium (Figure S3C). Furthermore, comparative proteomic analysis demonstrated reduced ubiquitination and total protein levels of both enzymes during infection (Figure S3D), indicating that *A. alternata* employs distinct mechanisms to regulate carbon metabolism.

Our study determined that UBPs function in colony growth, sporulation, and virulence of the *A. alternata* tangerine pathotype. Specifically, Aaubp14 regulates ACT biosynthesis and pathogenicity by modulating both secondary metabolism and pathogenicity-related protein expression through ubiquitination pathways, providing insights for developing effective control strategies against citrus brown spot disease.

## 5. Conclusions

Taken together, this is the first report on the function of UBP deubiquitinase in Dothideomycetes. Our study demonstrates that the AaUBPs play diverse roles in development and pathogenicity, with Aaubp14 essential for conidiation, ACT biosynthesis, and virulence. Integrated quantitative ubiquitome and proteome analyses suggest Aaubp14 promotes ACT biosynthesis through secondary metabolism regulation and metabolic enzyme stabilization, while modulating pathogenicity-related protein expression via ubiquitination control. These findings not only expand our understanding of the biological functions of the UBPs in *A. alternata* but also provide valuable insights into the complex ubiquitination-mediated regulatory mechanisms of Aaubp14, thereby enriching our knowledge of ubiquitination functions in fungi.

## Data Availability

The original data presented in the study are openly available in FigShare at https://doi.org/10.6084/m9.figshare.28926131 (accessed on 4 May 2025).

## Supplementary Materials

The following supporting information can be downloaded at https://www.mdpi.com/article/10.3390/jof11070495/s1, Figure S1: Deletion of 13 *Aaubp* genes; Figure S2: UHPLC-MS/MS analysis of chromatographic peak areas for five secondary metabolites in Z7 and Δ*Aaubp14* strains; Figure S3: Aaubp14 regulates carbon utilization; Table S1: Primers used in this study; Table S2: Proteomic data for previously reported pathogenicity-related proteins in *A. alternata* tangerine pathotype.
