# Supplementary material for: Functional Characterization of Deubiquitinase UBP Family and Proteomic Analysis of Aaubp14-Mediated Pathogenicity Mechanism in Alternaria alternata
Source: J Fungi (Basel). 2025 Jun 29;11(7):495. doi: 10.3390/jof11070495 (PMC12295320; doi:10.3390/jof11070495)
Supplement: Supplementary file 1 [file jof-11-00495-s001.zip › jof-3652568-supplementary.pdf]

Supplementary Materials

Supplementary Figures

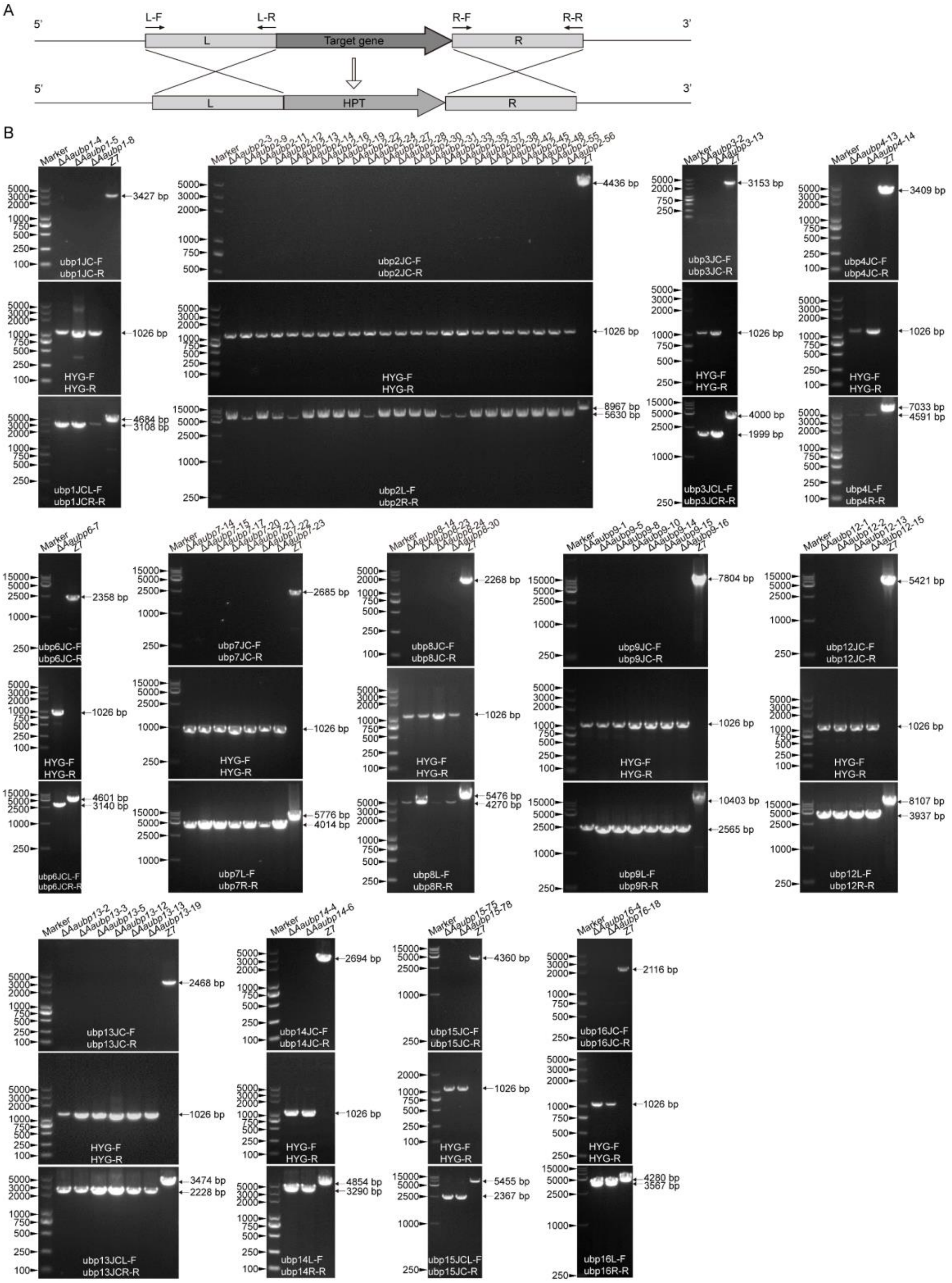

Figure S1. Deletion of 13 *Aaubp* genes. (A) Split-marker homologous recombination strategy for gene deletion. (B) PCR verification of individual gene deletion mutants by gel electrophoresis.

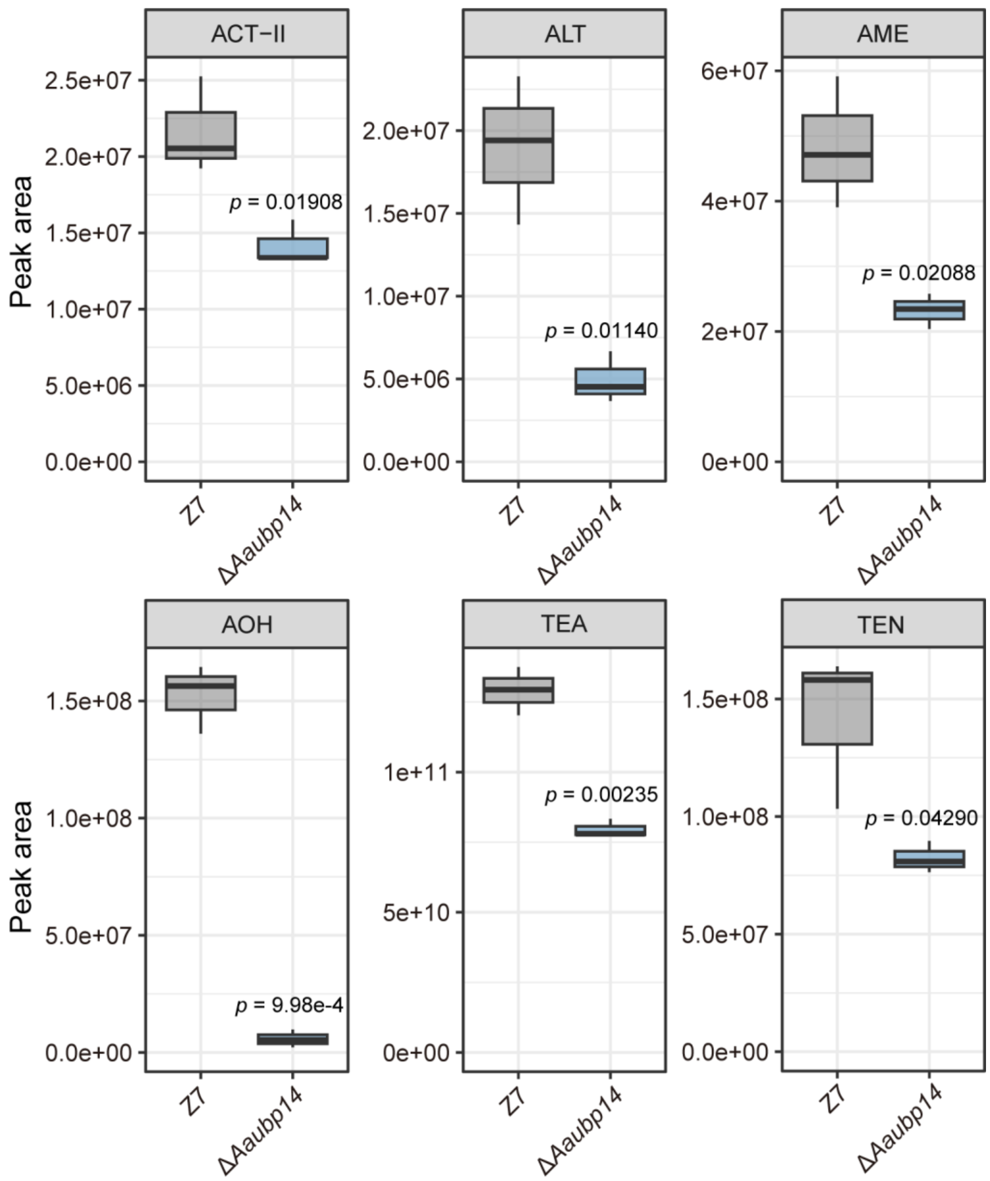

Figure S2. UHPLC-MS/MS analysis of chromatographic peak areas for five secondary metabolites in Z7 and Δ*Aaubp14* strains. Peak areas of Z7 were normalized based on the dry weight ratio of Z7 to Δ*Aaubp14* mycelial biomass. ALT, Alternuene; AME, Alternariol methyl ether; AOH, Alternariol; TEA, Tenuazonic acid; TEN, Tentoxin.

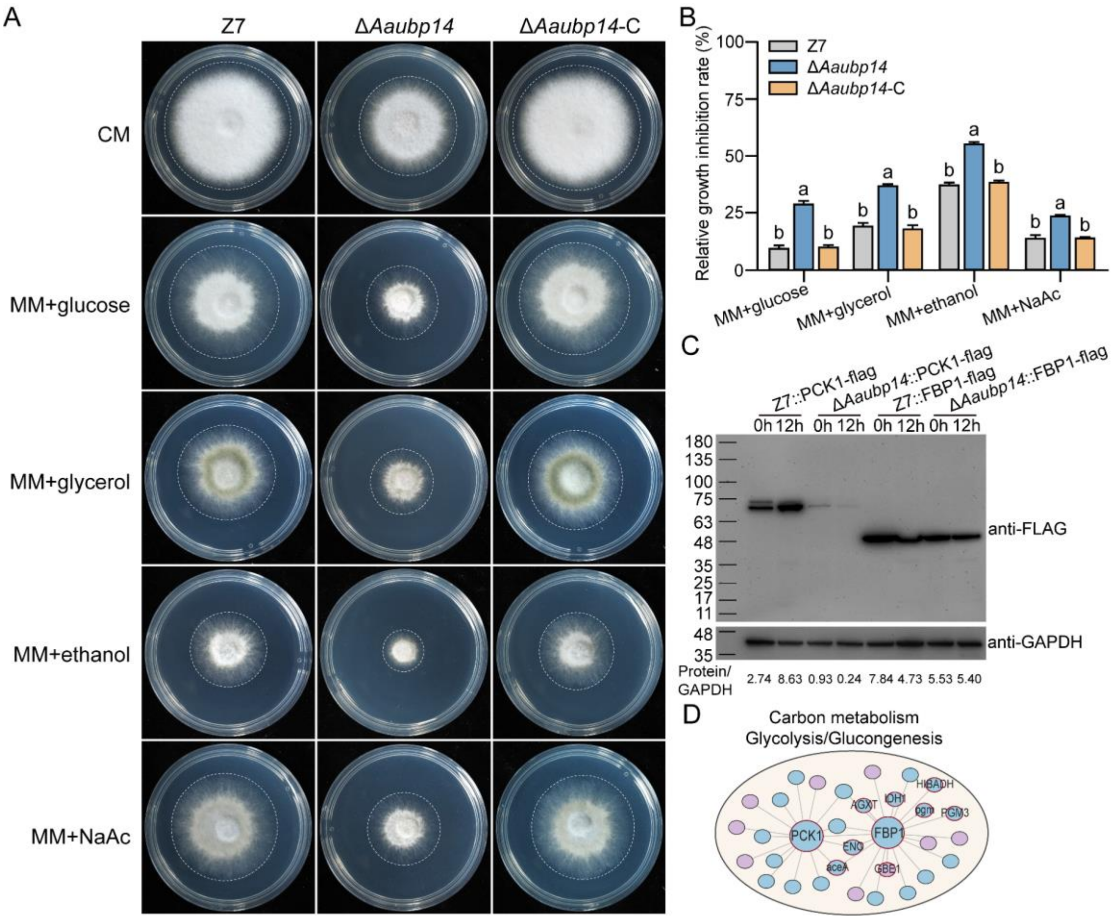

Figure S3. Aaubp14 regulates carbon utilization. (A) Colony morphology of Z7, Δ*Aaubp14*, and Δ*Aaubp14*-C after 72-h growth on CM or MM supplemented with 2% glucose, 2% glycerol, 2% ethanol, or 5 mM sodium acetate. Dashed circles indicate mycelial growth radius. (B) Growth inhibition analysis of Z7, Δ*Aaubp14*, and Δ*Aaubp14*-C on different carbon sources. Data are presented as mean ± SE. Different letters indicate significant differences at $P < 0.05$ determined by least significant difference (LSD) test. (C) Western blot analysis of FLAG-tagged PCK1 (70 kDa) and FBP1 (40 kDa) protein abundance in Z7 and Δ*Aaubp14*. Strains were initially cultured in MM with 5 mM sodium acetate, then transferred to glucose-supplemented MM and harvested at 0 and 12 hpi. Protein levels were normalized using GAPDH as the loading control. Relative protein abundance is indicated below each band. (D) Protein-protein interaction network analysis centered on PCK1 and FBP1. Proteomic analysis identified DEPs in Δ*Aaubp14* compared to Z7, represented by pink (increased) and blue (decreased) nodes. Red circles denote proteins with elevated ubiquitination levels in the Δ*Aaubp14* mutant.

Supplementary Tables

Table S1. Primers used in this study.

**Primer**	**Secquence (5′-3′)**	**Relevant characteristics**
Aaactin-RT	F: GGTATCTCCGACCGTATGCA<br>R: CTCTCGTCGTACTCCTGCTT	Expression detection of *Aaactin* by qRT-PCR
Aaubp1-RT	F: CTAAACCCGAATCGCCACTG<br>R: GCTGCTTCGGTTGATATGCA	Expression detection of *Aaubp1* by qRT-PCR
Aaubp2-RT	F: CTCTGGAGTCGGAAATCAACAC<br>R: TCGAGCACTATACCCTCCTCTA	Expression detection of *Aaubp2* by qRT-PCR
Aaubp3-RT	F: CCATTGACATTGAGAGCCCG<br>R: AAGTTCTTGAGGGCATCGGA	Expression detection of *Aaubp3* by qRT-PCR
Aaubp4-RT	F: TCGCCGAAATCATAGCCTCT<br>R: GGTAGGTATCAGTTCGGCGA	Expression detection of *Aaubp4* by qRT-PCR
Aaubp6-RT	F: GCGACCTCTTCAAGCAGATG<br>R: ATCTGTGACCAAGCCTCCTC	Expression detection of *Aaubp6* by qRT-PCR
Aaubp7-RT	F: CGAGAAGAATCACCGTATCTGC<br>R: ATCCGTTGCTGCTGTAGAATTC	Expression detection of *Aaubp7* by qRT-PCR
Aaubp8-RT	F: CGTTTTGTGGGAAGCTGTCA<br>R: TCTCCTTGACTGTGAAGCGT	Expression detection of *Aaubp8* by qRT-PCR
Aaubp9-RT	F: CTGGGACGACTTCTATCTCACA<br>R: TCACGATTCTCCATAGGTCAGG	Expression detection of *Aaubp9* by qRT-PCR

Aaubp12-RT	F: GAGGTTACAATGACGGATGCAA<br>R: GTGGTTTTGAGTAATCGACGGT	Expression detection of *Aaubp12* by qRT-PCR
Aaubp13-RT	F: CCGCCAAAATGACACAGGAA<br>R: ATGTTCGACTGGGTGATGGT	Expression detection of *Aaubp13* by qRT-PCR
Aaubp14-RT	F: TCAAATGCTATGAGTGCGGC<br>R: CAGAGTGTATGTTCGCAGGC	Expression detection of *Aaubp14* by qRT-PCR
Aaubp15-RT	F: TTCCTTCGGTTGGGATTCGA<br>R: ACCCGGGATGATTCGTAGTC	Expression detection of *Aaubp15* by qRT-PCR
Aaubp16-RT	F: AAGCCTCTTCCACCTCACTC<br>R: CGATTGTGCATCTGGGTCAG	Expression detection of *Aaubp16* by qRT-PCR
HYG	F: ATGAAAAAGCCTGAACTCAC<br>R: CTATTCCTTTGCCCTCGGAC	Amplification of *HYG* cassette
Aaubp1-L	F: AAGGGAAGAGTGACCGCAAG<br>R: GCTGCAGGAATTCGATATCAACGGAATTTTGGCGTTCACGA	Gene deletion vector construction of *Aaubp1*
HY-Aaubp1	F: TCGTGAACGCCAAAATTCCGTTGATATCGAATTCCTGCAGC<br>R: CTGCTCCATACAAGCCAACC	
YG-Aaubp1	F: TGTCCTGCGGGTAAATAGC<br>R: GTTCGCTTAACCCCATGCACGTCGGCATCTACTCTATTCCTTT	
Aaubp1-R	F: AAAGGAATAGAGTAGATGCCGACGTGCATGGGGTTAAGCGAAC<br>R: TTCCAAGAAGTGTGGCGAGG	
Aaubp1-JC	F: GTTTGCTCTGTTCGACCCCT<br>R: CTGTATGCGCTGGCGAAGTC<br>L-F: TTGGGGGAGAAGGGGGTAAT<br>R-R: CTGTATGCGCTGGCGAAGTC	PCR verification of *Aaubp1* deletion mutant

Aaubp2-L	F: GGTTGCGATGGTTGTTGTTCA<br>R: GCTGCAGGAATTCGATATCAAGCCGACTTCACCAACGTCTA	Gene deletion vector construction of *Aaubp2*
HY-Aaubp2	F: TAGACGTTGGTGAAGTCGGCTTGATATCGAATTCCTGCAGC<br>R: CTGCTCCATACAAGCCAACC	
YG-Aaubp2	F: TGTCCTGCGGGTAAATAGC<br>R: GGGTCACAAAAACAGTGCCCGTCGGCATCTACTCTATTCCTTT	
Aaubp2-R	F: AAAGGAATAGAGTAGATGCCGACGGGCACTGTTTTTGTGACCC<br>R: GGCTTGATCGTTGTTACGGC	
Aaubp2-JC	F: CCACCAGCGTGTGCTAACTA<br>R: AAGAGGCGGAGGAAGGAAGA	PCR verification of *Aaubp2* deletion mutant
Aaubp3-L	F: TGGTGTCAAAGCACAGCGG<br>R: GCTGCAGGAATTCGATATCAAGCGAGTTGGACCGCAAACC	Gene deletion vector construction of *Aaubp3*
HY-Aaubp3	F: GGTTTGCGGTCCAACTCGCTTGATATCGAATTCCTGCAGC<br>R: CTGCTCCATACAAGCCAACC	
YG-Aaubp3	F: TGTCCTGCGGGTAAATAGC<br>R: CTTCCCAGGTCGTAGATGCTGTCGGCATCTACTCTATTCCTTT	
Aaubp3-R	F: AAAGGAATAGAGTAGATGCCGACAGCATCTACGACCTGGGAAG<br>R: CGAGGTTGAGTTCCTTGGCT	
Aaubp3-JC	F: ACCTCATCCCAATCCACCCT<br>R: TGTGTGTATGTGAACGCTGG<br>L-F: GAAGGTGCAGAAGCCTTGGA<br>R-R: GGACGCGATGCATGTGATTG	PCR verification of *Aaubp3* deletion mutant
Aaubp4-L	F: CTTGCGTTGGAAGCCTGTTC<br>R: GCTGCAGGAATTCGATATCAATGCGTAGAGAGGCAGTGTTG	Gene deletion vector construction of *Aaubp4*

HY-Aaubp4	F: CAACACTGCCTCTCTACGCATTGATATCGAATTCCTGCAGC<br>R: CTGCTCCATACAAGCCAACC	
YG-Aaubp4	F: TGTCCTGCGGGTAAATAGC<br>R: AATAGAAGAGGGGTTGCGGCGTCGGCATCTACTCTATTCCTTT	
Aaubp4-R	F: AAAGGAATAGAGTAGATGCCGACGCCGCAACCCCTCTTCTATT<br>R: TCATCGGTCGCCTTCTAAGC	
Aaubp4-JC	F: ACACCACCATGAACGGCTAC<br>R: AGAGAGACCATCGTGAACCC	PCR verification of *Aaubp4* deletion mutant
Aaubp6-L	F: GTCGTCCTCTTCTCCGTTCC<br>R: GCTGCAGGAATTCGATATCAATGACACTCGCAGTCGCAATC	Gene deletion vector construction of *Aaubp6*
HY-Aaubp6	F: GATTGCGACTGCGAGTGTCATTGATATCGAATTCCTGCAGC<br>R: CTGCTCCATACAAGCCAACC	
YG-Aaubp6	F: TGTCCTGCGGGTAAATAGC<br>R: GAAACGCTGTGGTAGACGGAGTCGGCATCTACTCTATTCCTTT	
Aaubp6-R	F: AAAGGAATAGAGTAGATGCCGACTCCGTCTACCACAGCGTTTC<br>R: GTCGCTGTATCCTGAGCCC	
Aaubp6-JC	F: TAAACACTCTCTCGCCTCGC<br>R: ACTCCTGCGAGATTAGTTGCT<br>L-F: TCTACACAAATACACCAGTCAATCA<br>R-R: GGCGTCTTGCGAGTAACA	PCR verification of *Aaubp6* deletion mutant
Aaubp7-L	F: GACGAACCTGGAACACCCTT<br>R: GCTGCAGGAATTCGATATCAATTGTGTGTTTGGTTGGTGGG	Gene deletion vector construction of *Aaubp7*
HY-Aaubp7	F: CCCACCAACCAAACACACAATTGATATCGAATTCCTGCAGC<br>R: CTGCTCCATACAAGCCAACC	

YG-Aaubp7	F: TGTCCTGCGGGTAAATAGC<br>R: CTCCAACGCGCATCACAAATGTCGGCATCTACTCTATTCCTTT	
Aaubp7-R	F: AAAGGAATAGAGTAGATGCCGACATTTGTGATGCGCGTTGGAG<br>R: TGGCGTTTCTTGGAGACCTA	
Aaubp7-JC	F: GCTCTTTCGCTTCCGTCAAC<br>R: AACAAAACTGGGGACGGACC	PCR verification of *Aaubp7* deletion mutant
Aaubp8-L	F: GAATACGGGCACCTGGGTAG<br>R: GCTGCAGGAATTCGATATCAATTACCACCAAGGAAGACGCC	Gene deletion vector construction of *Aaubp8*
HY-Aaubp8	F: GGCGTCTTCCTTGGTGGTAATTGATATCGAATTCCTGCAGC<br>R: CTGCTCCATACAAGCCAACC	
YG-Aaubp8	F: TGTCCTGCGGGTAAATAGC<br>R: TGCGTGAAAACAAGTGTCGCGTCGGCATCTACTCTATTCCTTT	
Aaubp8-R	F: AAAGGAATAGAGTAGATGCCGACGCGACACTTGTTTTCACGCA<br>R: GGTAGGTTACTTTGCTCCCCC	
Aaubp8-JC	F: CGCCTGTGCTGATTGGTTTC<br>R: GAGGCGACTGATGGAGAAGG	PCR verification of *Aaubp8* deletion mutant
Aaubp9-L	F: ATGTGTAGGCTGGATGCGAG<br>R: GCTGCAGGAATTCGATATCAAGTTGGGTCTCATCTGCGTCA	Gene deletion vector construction of *Aaubp9*
HY-Aaubp9	F: TGACGCAGATGAGACCCAACTTGATATCGAATTCCTGCAGC<br>R: CTGCTCCATACAAGCCAACC	
YG-Aaubp9	F: TGTCCTGCGGGTAAATAGC<br>R: TCCCCAAAAACCGAAGTCCAGTCGGCATCTACTCTATTCCTTT	
Aaubp9-R	F: AAAGGAATAGAGTAGATGCCGACTGGACTTCGGTTTTTGGGGA<br>R: GCGACTTCAAAATGTAATTGGCA	

Aaubp9-JC	F: GCTGCTGTTGCCTGTACTTG<br>R: GTTCGATCTCCTCAGCCCAC	PCR verification of *Aaubp9* deletion mutant
Aaubp12-L	F: CGTGCCTATCTCAGCGTCTT<br>R: GCTGCAGGAATTCGATATCAAAGCCTCGCTCAACAGTCATT	Gene deletion vector construction of *Aaubp12*
HY-Aaubp12	F: AATGACTGTTGAGCGAGGCTTTGATATCGAATTCCTGCAGC<br>R: CTGCTCCATACAAGCCAACC	
YG-Aaubp12	F: TGTCCTGCGGGTAAATAGC<br>R: CCTTTTTCCATCGCCAAGCCGTCGGCATCTACTCTATTCCTTT	
Aaubp12-R	F: AAAGGAATAGAGTAGATGCCGACGGCTTGGCGATGGAAAAAGG<br>R: CGAGAGCGGAGCAAGAGTAG	
Aaubp12-JC	F: CACCCTTTCTCAGCACAGCA<br>R: ATGAACGCGACCTGTACACT	PCR verification of *Aaubp12* deletion mutant
Aaubp13-L	F: GGGTGAATGAGAAGTCGCGT<br>R: GCTGCAGGAATTCGATATCAAGAGATGTCAGGAGCGAAGGG	Gene deletion vector construction of *Aaubp13*
HY-Aaubp13	F: CCCTTCGCTCCTGACATCTCTTGATATCGAATTCCTGCAGC<br>R: CTGCTCCATACAAGCCAACC	
YG-Aaubp13	F: TGTCCTGCGGGTAAATAGC<br>R: ACGCTTCGGTAGGAAGACTGGTCGGCATCTACTCTATTCCTTT	
Aaubp13-R	F: AAAGGAATAGAGTAGATGCCGACCAGTCTTCCTACCGAAGCGT<br>R: AGGACCCCGCAGTACAAAAG	
Aaubp13-JC	F: GGTGCTGTAAGTCCCCAAGA<br>R: CAAAAGCCACCCTGTTTGACA<br>L-F: GAAGCCAGCAGATGTGAGGT<br>R-R: ACACACCACCCATCCTTGAG	PCR verification of *Aaubp13* deletion mutant
Aaubp14-L	F: ATGGGGAAGAGTCGAACAGC<br>R: GCTGCAGGAATTCGATATCAAGCCCGACTTGATACACGCTT	Gene deletion vector construction of *Aaubp14*

HY-Aaubp14	F: AAGCGTGTATCAAGTCGGGCTTGATATCGAATTCCTGCAGC<br>R: CTGCTCCATACAAGCCAACC	
YG-Aaubp14	F: TGTCCTGCGGGTAAATAGC<br>R: GACGAGCGAACATCAGTTGCGTCGGCATCTACTCTATTCCTTT	
Aaubp14-R	F: AAAGGAATAGAGTAGATGCCGACGCAACTGATGTTCGCTCGTC<br>R: TCTTCAGCAAGCATGGAGCA	
Aaubp14-JC	F: TTCTGGTAGTGGCGGTAGTG<br>R: CTGCCTTAGCGACCTTCTC	PCR verification of *Aaubp14* deletion mutant
Aaubp15-L	F: ATCTAGGCGTTGTTGCACCT<br>R: GCTGCAGGAATTCGATATCAAATCGTTGGGTTCCTGCACTT	Gene deletion vector construction of *Aaubp15*
HY-ubp15	F: AAGTGCAGGAACCCAACGATTTGATATCGAATTCCTGCAGC<br>R: CTGCTCCATACAAGCCAACC	
YG-ubp15	F: TGTCCTGCGGGTAAATAGC<br>R: TGCGCCATGTAAGTTGGGATGTCGGCATCTACTCTATTCCTTT	
Aaubp15-R	F: AAAGGAATAGAGTAGATGCCGACATCCCAACTTACATGGCGCA<br>R: GGGTCTCGACAACTGGATGG	
Aaubp15-JC	F: CTCATCCTTACCCGCATCCC<br>R: CACCGCTTCTACCTGGGAAC<br>L-F: CACCAATAGCAAGACGGGGT	PCR verification of *Aaubp15* deletion mutant
Aaubp16-L	F: CGATCCCTTCGCTCTGATCC<br>R: GCTGCAGGAATTCGATATCAAGAGCCTACGGCAAGTAGCAC	Gene deletion vector construction of *Aaubp16*
HY-Aaubp16	F: GTGCTACTTGCCGTAGGCTCTTGATATCGAATTCCTGCAGC<br>R: CTGCTCCATACAAGCCAACC	

YG-Aaubp16	F: TGTCCTGCGGGTAAATAGC<br>R: ACTTCGAGATGCAGAGTCGCGTCGGCATCTACTCTATTCCTTT	
Aaubp16-R	F: AAAGGAATAGAGTAGATGCCGACGCGACTCTGCATCTCGAAGT<br>R: TACGCCCATGTCAGCGATAC	
Aaubp16-JC	F: CCTCACGAGCAAACGACAGA<br>R: CCCGCCACTGTCTGTACAAT	PCR verification of *Aaubp16* deletion mutant
ACTT1-RT	F: CAGGGTCAGTTGGGTGTCTT<br>R: GTTGGGGGAAAGGCATATTT	Expression detection of *ACTT1* by qRT-PCR
ACTT2-RT	F: CTTCATTCGCTCTGGAAAGG<br>R: TGGCACAATTCTTTCATCCA	Expression detection of *ACTT2* by qRT-PCR
ACTT3-RT	F: GTTTTTACTGGCGAGGGACA<br>R: CGGATACTCATTGGCAAGGT	Expression detection of *ACTT3* by qRT-PCR
ACTT5-RT	F: AAGCTTGAGCCTTTCCTCGC<br>R: CCTGAAACCGCTGTCCTCAC	Expression detection of *ACTT5* by qRT-PCR
ACTT6-RT	F: CTCACGGGAAAAGGCAAATA<br>R: TCGATGGCTCCAAATAGGTC	Expression detection of *ACTT6* by qRT-PCR
ACTTS1-RT	F: AGCCTCACAAGCCAATGTCG<br>R: GCGGCAGGGTGATATGGTTT	Expression detection of *ACTTS1* by qRT-PCR
ACTTS2-RT	F: ACTACAGGGACCCCGAATGC<br>R: AGCAGCGCAGTGTATTTCCC	Expression detection of *ACTTS2* by qRT-PCR
ACTTS3-RT	F: GCCTGTTACCGTCAAGCCTC<br>R: TACGGCTGGTGAGCACTACA	Expression detection of *ACTTS3* by qRT-PCR

ACTTS4-RT	F: GCGACCCAGAGAAAACATCC<br>R: GCTGTCCACGAATCTTGACC	Expression detection of *ACTTS4* by qRT-PCR
ACTTR-RT	F: GGGTTGACATTTTCGGGACTAG<br>R: ATGCTAAAGACAACCTCCTCCA	Expression detection of *ACTTR* by qRT-PCR
Aaubp14C	Pro-F: GGTACCCGGGGATCCTCTAGACGCAGATGAGCGAAAGTCTAA<br>Pro-R: GTTCACGTGTGTGCAAGCCATGCTTGGTGGAGTTTGCAGGGA<br>F: TCCCTGCAAACTCCACCAAGCATGGCTTGCACACACGTGAAC<br>R: CTCCTCGCCCTTGCTCACCATCAAACGCCTGAAGAAATACAC	Amplification of complete *Aaubp14* and its native promoter for construction of the vector for complementation
PtrpC-PCK1<br><br>PCK1<br><br>Flag-PCK1	F: GGTACCCGGGGATCCTCTAGATTGATATCGAATTCCTGC<br>R: TTTAATACCATTCGAAGCCATTTGGATGCTTGGTAGAATAGG<br>F: CCTATTCTACCAAGCATCCAAATGGCTTCGAATGGTATTAAA<br>R: CGTCATGGTCTTTGTAGTCGTGCTTGGGGCAGCAGCATAC<br>F: GTATGCTGCTGCCCCAAGCACGACTACAAAGACCATGACG<br>R: ACGAAAGCTCTGCAGGTCGACACTCCTCCGCTTCAAGTC	Amplification of TrpC promoter, complete *PCK1*, and flag tag for construction of the vector for carbon source utilization study
PtrpC-FBP1<br><br>FBP1<br><br>Flag-FBP1	F: GGTACCCGGGGATCCTCTAGATTGATATCGAATTCCTGC<br>R: CTCGTTACTGTTGGAAGCCATTTGGATGCTTGGTAGAATAGG<br>F: CCTATTCTACCAAGCATCCAAATGGCTTCCAACAGTAACGAG<br>R: CGTCATGGTCTTTGTAGTCTTTCTTGTAGTTCTTGTGCGC<br>F: GCGCACAAGAACTACAAGAAAGACTACAAAGACCATGACG<br>R: ACGAAAGCTCTGCAGGTCGACACTCCTCCGCTTCAAGTC	Amplification of TrpC promoter, complete *FBP1*, and flag tag for construction of the vector for carbon source utilization study

Table S2. Proteomic data for previously reported pathogenicity-related proteins in *A. alternata* tangerine pathotype.

Protein ID	Protein name	Δ*Aaubp14*/WT Ratio	*p*-value	Regulated level	Ubiquitinated level	Reference
A0A177D4K0	Atg1, non-specific serine/threonine protein kinase	2.848086676	5.42217E-05	Up	Down	Wu et al., 2024
J7G926	PKAr, cAMP-dependent protein kinase regulatory subunit	2.629227293	1.4819E-05	Up	Normal	Tsai et al., 2013
A0A177DN73	Hos2, histone deacetylase	2.505103065	0.004434929	Up	Normal	Ma et al., 2021
A0A177DTL1	RTT109, histone acetyltransferase	2.397454072	0.052594601	Normal	Normal	Ma et al., 2021
A0A177E010	ubp6, deubiquitinase	2.341605362	1.10863E-06	Up	Down	This study
A0A177DI99	SSK1, response regulatory domain-containing protein	2.260685537	0.018050841	Up	Normal	Yu et al., 2016
A0A177DF74	Rpd3, histone deacetylase	1.933744145	7.26903E-05	Up	Up	Ma et al., 2021
A0A177D9W0	Cut2, Cutinase	1.872400888	0.000316126	Up	Normal	Fu et al., 2020
A0A177D9W0	Cut15, Cutinase	1.872400888	0.000316126	Up	Normal	Fu et al., 2020
D3J126	HogA, mitogen-activated protein kinase	1.822861706	1.01329E-05	Up	Up	Lin and Chung, 2010
A0A177DTK6	Ghd2, histone demethylases	1.805219987	7.37518E-05	Up	Normal	Meng et al., 2022
A0A177DFP3	PLC1, phosphoinositide phospholipase C	1.796380276	0.005647832	Up	Down	Tsai and Chung, 2014
A0A177DV61	Hnrnp, histone methyltransferases	1.769021016	4.10021E-05	Up	Down	Meng et al., 2022
A0A177DU44	Csn5, COP9 signalosome complex subunit 5	1.708178142	0.009418987	Up	Normal	Wang et al., 2018
A0A177E1W0	ubp12, deubiquitinase	1.700342265	0.001825015	Up	Normal	This study

A0A177E2I1	ubp9, deubiquitinase	1.660062628	0.190126207	Normal	Normal	This study
A0A177DKN6	SIP2, β-subunit of Sucrose Non-Fermenting (SNF1) protein kinase	1.614536817	0.197013791	Normal	Normal	Tang et al., 2018
A0A173G6N8	Nac1, nascent polypeptide-associated complex subunit alpha	1.598385068	0.021082656	Up	Down	Wang et al., 2020
A0A177D2C0	Cut7, Cutinase	1.574487779	0.000164985	Up	Normal	Ma et al., 2019
A0A177D1M4	ubp3, deubiquitinase	1.529163167	0.015510451	Up	Normal	This study
A0A177DKG1	Ste12, STE-domain-containing protein	1.50774102	0.229273	Normal	Normal	Ma et al., 2019
A0A177DPT2	Gcn5, histone acetyltransferase	1.487916749	0.076721889	Normal	Normal	Ma et al., 2021
A0A177DCY5	StuA, Apses-domain-containing protein	1.450193689	0.014103466	Up	Down	Chen et al., 2023
A0A177DRE3	Trr1, thioredoxin reductase	1.44482092	0.006245255	Up	Down	Ma et al., 2018
A0A177E040	SNF1, α-subunit of Sucrose Non-Fermenting (SNF1) protein kinase	1.350844337	0.00940994	Up	Normal	Tang et al., 2020
A0A177DCZ9	Set2, histone methyltransferases	1.344727705	0.035693348	Up	Normal	Meng et al., 2022
A0A177DAJ6	FUS3, mitogen-activated protein kinase	1.329129153	0.000847947	Up	Down	Cho et al., 2007; Lin et al., 2010
A0A177DGY9	Pdel, Cyclic di-GMP phosphodiesterase PdeL	1.298408508	2.72583E-06	Up	Up	Lv et al., 2020
A0A177DDR5	SNF4, CBS-domain-containing protein	1.291772498	0.065376743	Normal	Normal	Tang et al., 2020
A0A177DHG5	Tfb5, General transcription and DNA repair factor IIH subunit TFB5	1.26665798	0.2726121	Normal	Normal	Fu et al., 2020

A0A177DBV0	Prb1, Subtilisin-like serine protease	1.24473572	0.002404489	Up	Up	Fu et al., 2020
A0A177DUJ3	ubp15, deubiquitinase	1.224655283	0.000367605	Up	Up	This study
A0A177D7P9	Sas3, histone acetyltransferase	1.219728003	0.017739295	Up	Normal	Ma et al., 2021
A0A177DER1	Gpx3, glutathione peroxidase	1.151163674	0.454807927	Normal	Down	Yang et al., 2015
A0A177D640	Pep4, Vacuolar protease A	1.142142698	0.000462336	Normal	Up	Fu et al., 2020
A0A177DP23	ubp7, deubiquitinase	1.135993479	0.379554622	Normal	Down	This study
A0A177DG15	ubp4, deubiquitinase	1.135280784	0.010351193	Normal	Normal	This study
A0A177D8R2	ubp13, deubiquitinase	1.120424093	0.733611613	Normal	Normal	This study
D3J127	SLT2, mitogen-activated protein kinase	1.097211202	0.013274939	Normal	Up	Yago et al., 2011
A0A177DXL7	ubp2, deubiquitinase	1.089583777	0.045523258	Normal	Up	This study
A0A177DT60	BioB, biotin synthase	1.051611075	0.221934171	Normal	Normal	Wu et al., 2020
A0A177DLX8	pex3, Peroxin-3	1.042956353	0.441159517	Normal	Up	Choo et al., 2023
A0A177E2S2	Tsa1, thioredoxin-dependent peroxiredoxin	0.988992338	0.655478014	Normal	Up	Ma et al., 2018
A0A177DGB3	Glr1, glutathione reductase	0.978597631	0.230002362	Normal	Up	Ma et al., 2018
A0A177DD11	Atg8, Autophagy-related protein	0.97303468	0.64542802	Normal	Down	Fu et al., 2020; Wu et al., 2022
U3Q4A0	CAL1, calcineurin phosphatase	0.877973172	0.003815177	Normal	Down	Tsai and Chung, 2014
A0A177DP18	Elp3, Elongator complex protein 3	0.869779557	0.054340507	Normal	Up	Ma et al., 2021
